# Functional Principal Component Analysis for Continuous Non‐Gaussian, Truncated, and Discrete Functional Data

**DOI:** 10.1002/sim.10240

**Published:** 2024-10-23

**Authors:** Debangan Dey, Rahul Ghosal, Kathleen Merikangas, Vadim Zipunnikov

**Affiliations:** ^1^ National Institute of Mental Health Bethesda Maryland USA; ^2^ Department of Epidemiology and Biostatistics University of South Carolina Columbia South Carolina USA; ^3^ Department of Biostatistics Johns Hopkins Bloomberg School of Public Health Baltimore Maryland USA

**Keywords:** covariance estimation, discrete functional data, EMA, functional data analysis, Gaussian copula

## Abstract

Mobile health studies often collect multiple within‐day self‐reported assessments of participants' behavior and well‐being on different scales such as physical activity (continuous scale), pain levels (truncated scale), mood states (ordinal scale), and the occurrence of daily life events (binary scale). These assessments, when indexed by time of day, can be treated and analyzed as functional data corresponding to their respective types: continuous, truncated, ordinal, and binary. Motivated by these examples, we develop a functional principal component analysis that deals with all four types of functional data in a unified manner. It employs a semiparametric Gaussian copula model, assuming a generalized latent non‐paranormal process as the underlying generating mechanism for these four types of functional data. We specify latent temporal dependence using a covariance estimated through Kendall's τ bridging method, incorporating smoothness in the bridging process. The approach is then extended with methods for handling both dense and sparse sampling designs, calculating subject‐specific latent representations of observed data, latent principal components and principal component scores. Simulation studies demonstrate the method's competitive performance under both dense and sparse sampling designs. The method is applied to data from 497 participants in the National Institute of Mental Health Family Study of Mood Spectrum Disorders to characterize differences in within‐day temporal patterns of mood in individuals with the major mood disorder subtypes, including Major Depressive Disorder and Type 1 and 2 Bipolar Disorder. Software implementation of the proposed method is provided in an R‐package.

## 1 Introduction

**FIGURE 1:**
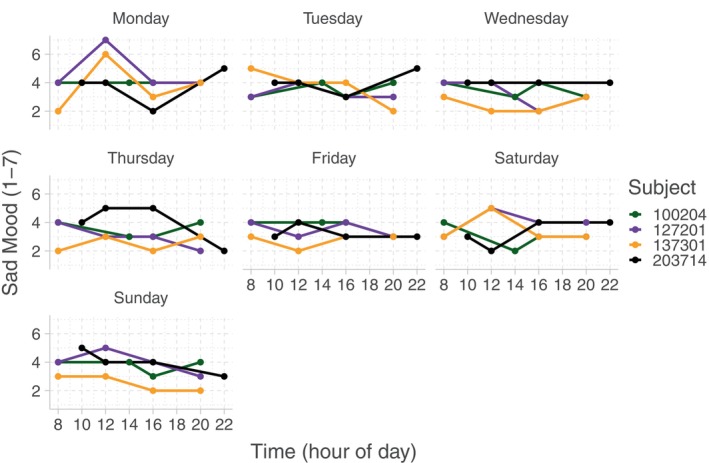
Within‐day temporal pattern of mood ratings (1 = Most Happy to 7 = Most Sad, 4 = Neutral) over 7 days for four different subjects in National Institute of Mental Health Family Study of Mood Disorders Subtypes.

Multiple real‐time assessments of human physical and mental experiences and health‐related behaviors have recently been made possible through mobile digital health monitoring (mHealth). Intensive longitudinal data collected with mHealth tools, including wearables and smartphone apps, allows researchers to better understand within‐day temporal patterns of experiences and behaviors and their influence on health. National Institute of Mental Health Family Study of the Mood Spectrum Disorders employed an mHealth app that collected multiple within‐day participants' reports on levels of sadness/happiness, energy, anxiety, pain, levels of physical activity, and many other experiences and behaviors. Self‐reported mood was reported on a Likert ordinal scale of (1) to (7) (very happy to very sad, with (4) = neutral). Figure 1 displays diurnal

(within‐day) mood trajectories over four time‐points over 7 days of the week for four randomly chosen participants.

The study also collected self‐reports on binary (negative encounters: present/not present), truncated (pain: yes/no, and if yes, what is the level of pain on the scale 1–10), and multiple continuous scales covering quality and duration of sleep, time spent in physical activity of different intensity (light, moderate‐to‐vigorous). Indexed by time‐of‐day, these assessments can be treated as functional observations of continuous non‐Gaussian, truncated, ordinal, and binary types. Figure 1 illustrates typical analytical challenges for these types of functional data: (i) they are sparsely observed, (ii) observation points can be highly irregular, and (iii) there exists heterogeneity in subject‐specific interpretations of Likert scales (specifically, for truncated, ordinal, and continuous scales). In this paper, we develop a covariance estimation and principal component analysis that can treat functional data of four types, including continuous non‐Gaussian, truncated, ordinal, and binary, in a unified way and address the above‐mentioned statistical challenges. Next, we provide a review of relevant methods and place our work within existing literature.

The early developments in functional principal component analysis (FPCA) [1, 2, 3, 4, 5, 6] primarily focused on continuous Gaussian functional data collected under sparse or dense designs. These methods rely on smoothing noisy sample covariances [3, 7, 8] or pre‐smoothing the sample of functions and then diagonalizing the sample covariance function of the smoothed curves [1, 5]. There are some interesting recent developments in FPCA for continuous non‐Gaussian and non‐continuous functional data. Proposed approaches mostly extend FPCA to continuous and non‐continuous types generated from generalized exponential family via generalized linear models. The original work of Hall, Müller, and Yao [9] relies on a known link function connecting observed generalized outcome to a latent continuous outcome and proceeds with the covariance estimation of the functional latent process. These models have been extended further to accommodate conditional function‐on‐scalar regression models through frequentist [10, 11, 12] and Bayesian paradigms [13, 14]. Based on the registration approach for exponential family, Wrobel et al. [15] released an R package *BFPCA* that can perform a generalized FPCA of binary functional data. Li, Staudenmayer, and Carroll [16] proposed to model multiple functional outcomes jointly and developed a hierarchical method for modeling paired functional data consisting of simultaneous measurements of a continuous and a binary variable. This approach modeled the correlation between the paired variables by the correlation across the principal component scores of latent “pseudo” normal distributions. All these approaches focus primarily on binary and count data and rely either on known link functions or model‐based likelihood. Recently, Leroux, Crainiceanu, and Wrobel [17] developed a fast generalized FPCA that smooths local generalized linear models and requires dense functional data.

In a seemingly related approach to our proposal, but conceptually different, Zhong et al. [18] developed a robust FPCA method based on the Kendall's τ function for continuous non‐Gaussian functional data. Compared to our proposal, they solely focus on continuous functional data and use a nonparametric kernel‐based estimation method. Solea and Li [19] introduced a functional copula Gaussian graphical model for multivariate continuous functional data while assuming a nonparanormal distribution (Definition 1) on the coefficients of the Karhunen–Loeve expansions. Under nonparanormal distributional assumptions, non‐Gaussian continuous functional data can also be transformed to Gaussian functional data [20, 21] to perform FPCA under normality assumptions. Although these approaches are robust and semiparametric, they are restricted to continuous functional data and cannot be applied to truncated, ordinal, or binary functional data. We propose a unified FPCA for continuous non‐Gaussian, truncated, ordinal, and binary functional data. The approach is rank‐based and is, hence, invariant to arbitrary monotone transformations (and addresses the subject‐specific interpretation of reporting scales in mHealth applications) and robust. Importantly, the method can accommodate both functional data collected under both dense and sparse designs.

Our covariance estimation and principal component analysis approach is based on semiparametric Gaussian Copula (SGC). Beyond being applied to handle continuous and multivariate functional data [19, 22], latent SGC [20, 23, 24, 25, 26, 27, 28, 29] are powerful for modeling all four types (continuous, truncated, ordinal, and binary) of data and provide scale‐free, robust and fast algorithms. Here, we extend this framework to univariate functional data of continuous non‐Gaussian, truncated, or discrete type. Our key methodological contributions are: (i) defining a unifying data‐generating mechanism for the observed continuous, truncated or discrete functional data via a continuous Generalized Latent Nonparanormal process, (ii) developing a robust semiparametric method for estimating the smooth covariance of the latent Gaussian process based on the rank correlation (Kendall's τ) of the observed data with smoothing incorporated into bridging, (iii) covering both dense and sparse sampling designs, and (iv) calculating subject‐specific latent representations of observed data, latent principal components and latent principal component scores. By combining (i)–(iv), the proposed framework handles all four functional data types in a unified way. For continuous, truncated, and ordinal scales, the approach is scale‐invariant. This additionally addresses the problem of subject‐specific interpretability of Likert scales widely used by mHealth apps.

The rest of this article is organized as follows. Section 2 presents our modeling framework and illustrates the proposed covariance estimation method. In Section 3, we evaluate the performance of the proposed method via simulations and compare it with available existing methods of covariance estimation. Section 4 applies the method to the mHealth data from the NIMH Family Study. Section 5 presents concluding remarks and discusses possible extensions of this work.

## 2 Modeling Framework

We consider univariate functional data $X_{i}\left(t\right)$, for subjects i=1,2,…,n, where $X_{i}\left(t\right)$ denotes the functional observation for subject i and can be binary, ordinal, truncated or continuous. In this section, we assume that functional objects are observed on a dense and regular grid of points $S=\left\{t_{1},t_{2},\ldots ,t_{m}\right\}\subset 𝒯=\left[0,1\right]$. The proposed method is later extended to a more general set‐up of sparse and irregularly observed non‐Gaussian, truncated, and discrete functional data. Functional observations $X_{i}\left(\cdot \right)$ are assumed to be independent and identically distributed (i.i.d.) copies of X(⋅), an underlying smooth stochastic process. As in the case of continuous functional data, a major question of interest is estimating the covariance kernel of the process X(⋅). We propose using a SGC model [25, 29] for covariance modeling of continuous, truncated, or discrete‐type functional data. Next, we introduce the key components of the model.

Definition 1 (Nonparanormal distribution) A random vector $W={\left(W_{1},\ldots ,W_{p}\right)}^{\prime }$ follows a non‐paranormal distribution denoted as $W∼{\mathrm{NPN}}_{p}\left(0,\sum ,f\right)$, if there exists monotone transformation functions $f=\left(f_{1},\ldots ,f_{p}\right)$ such that $L=f\left(W\right)=\left(f_{1}\left(W_{1}\right),\ldots ,f_{p}\left(W_{p}\right)\right)∼N\left(0,\sum \right)$, with $\sum_{\mathrm{jj}}=1$ for 1≤j≤p. The constraints on diagonal elements of ∑ are made to ensure the identifiability of the distribution as demonstrated in Liu et al. [20] and Fan et al. [24].

Definition 2 (Generalized Latent Nonparanormal Process) For observed functional data X(t), we assume that there exists a latent process Z(t) that serves as a data generating mechanism for observed X(t) as follows:

(1) $$\begin{array}{ll} X\left(t\right) & =Z\left(t\right)\left(\text{continuous scale}\right),\text{or}\\ X\left(t\right) & =Z\left(t\right)I\left(Z\left(t\right)>\Delta \left(t\right)\right),\left(\text{truncated scale}\right),\text{or}\\ X\left(t\right) & =\sum_{k=0}^{l-1}\mathrm{kI}\left(\Delta_{k}\left(t\right)\leq Z\left(t\right)<\Delta_{k+1}\left(t\right)\right),\\ -\infty & =\Delta_{0}\left(t\right)\leq \Delta_{1}\left(t\right)\leq \cdots \leq \Delta_{l}\left(t\right)=\infty ,\left(\text{ordinal scale}\right),\text{or}\\ X\left(t\right) & =I\left(Z\left(t\right)>\Delta \left(t\right)\right),\left(\text{binary scale}\right) \end{array}$$

Suppose that for any finite set of points $S=\left\{t_{1},t_{2},\ldots ,t_{m}\right\},\left(\left(Z\left(t_{1}\right)\right),\cdots ,\left(Z\left(t_{m}\right)\right)\right)∼{\mathrm{NPN}}_{m}\left(0,C\left(S,S\right),f\right)$, where $f=(f_{t_{1}},\cdots ,f_{t_{m}})$ is a collection of m monotone transformation functions and ${\left(C\left(S,S\right)\right)}_{jj^{\prime }}=C\left(t_{j},t_{j^{\prime }}\right)$ for a scaled covariance function C:𝒯×𝒯↦[−1,1] with C(t,t)=1. Further, we denote V(t) as the latent multivariate normal process, that is, $V\left(t\right)=f_{t}\left(Z\left(t\right)\right)$ and $\left(V\left(t_{1}\right),\cdots ,V\left(t_{m}\right)\right)∼N\left(0,C\left(S,S\right)\right)$. Then, X(t) is defined as the Generalized Latent Nonparanormal Process (GLNPP) with the latent covariance function C and the cutoff process Δ(t).

The Definition 2 characterizes the GLNPP by describing the point‐wise distribution of a finite collection of time points. However, this initial definition does not immediately reveal whether this point‐wise characterization results in a stochastic process spanning an uncountable domain 𝒯. We can gain further insight by realizing that the finite‐dimensional realization of Z(t) is a Gaussian copula. Hence, we can express $f_{t}$ as $f_{t}=\Phi^{-1}\left(G_{t}\left(Z_{t}\right)\right)$, where $G_{t}$ represents the pointwise cumulative distribution function (CDF) for the marginal distribution of Z(t) and Φ denotes the standard Normal CDF [20]. Drawing upon Corollary 5.11 from Schmitz [30], we can ascertain that Z(⋅) constitutes a valid stochastic process across the entire domain. Given that Δ(⋅) is defined as a legitimate stochastic process, we can consequently affirm that X(⋅) also qualifies as a valid process spanning the entirety of the domain.

### 2.1 Covariance Estimation

**FIGURE 2:**
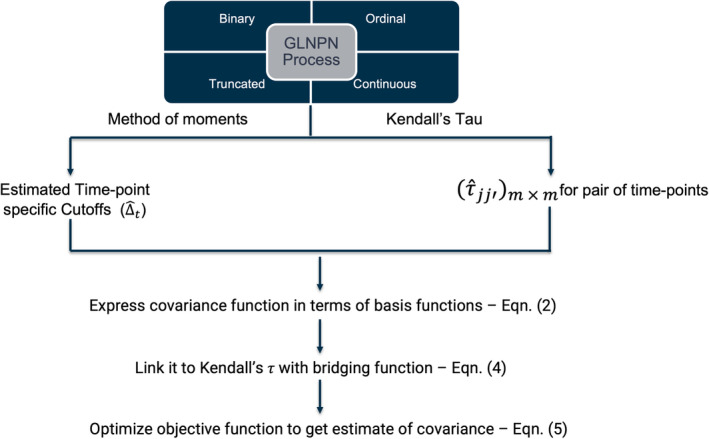
Stepwise estimation of the covariance function using observed data generated by GLNPN process.

We present the schematic representation of the steps needed to estimate the latent covariance function in Figure 2. The detailed steps are described below. We model the scaled covariance function C (note that C(t,t)=1 by definition) of the latent process as a tensor product spline on 𝒯×𝒯

(2) $$C\left(s,t\right)=g\left(\sum_{k=1}^{d}\sum_{l=1}^{d}u_{\mathrm{kl}}B_{k}\left(s\right)B_{l}\left(t\right)\right),s\neq t$$

Here, $B_{1}\left(\cdot \right),\ldots ,B_{d}\left(\cdot \right)$ is a collection of basis functions on 𝒯. In this article, we use cubic B‐spline basis functions; other basis functions can also be used depending on the problem of interest. Here $U={\left(u_{\mathrm{kl}}\right)}_{1\leq k\leq d,1\leq l\leq d}$ denotes the unknown coefficient matrix and $g=\frac{e^{x}-1}{e^{x}+1}$ is an inverse Fischer transformation to ensure the correlation values are within [−1,1]. We also enforce the constraint $u_{\mathrm{kl}}=u_{\mathrm{lk}}$ on the coefficient matrix U to ensure C(s,t)=C(t,s). Ideally, if we had observed the latent continuous process V(⋅), we would like to find U that minimizes the following nonlinear least square objective function:

(3) $$\begin{array}{l} l=\sum_{i=1}^{n}\sum_{1\leq j<j^{\prime }\leq m}{\left(V_{i}\left(t_{j}\right)V_{i}\left(t_{j^{\prime }}\right)-C\left(t_{j},t_{j^{\prime }}\right)\right)}^{2}\\ =\sum_{i=1}^{n}\;\sum_{1\leq j<j^{\prime }\leq m}{\left(V_{i}\left(t_{j}\right)V_{i}\left(t_{j^{\prime }}\right)-g\left(\sum_{k=1}^{d}\sum_{l=1}^{d}u_{\mathrm{kl}}B_{k}\left(t_{j}\right)B_{l}\left(t_{j^{\prime }}\right)\right)\right)}^{2} \end{array}$$

For finite‐dimensional multivariate distribution, the trick to estimate latent correlation $C\left(t_{j},t_{j^{\prime }}\right)$ is to use Kendall's τ rank correlation [24, 25, 26, 28] for the observed data and then bridge it to the corresponding latent correlation. This approach works because Kendall's τ is invariant to monotone transformations. Hence, we can express the objective function in (3) in terms of observed process X(⋅) and the Kendall's τ correlation. Next, we will give an overview of the Kendall's τ correlation and then come back to our discussion of the modified objective function in Section 2.1.2.

#### 2.1.1 Kendall's τ Rank Correlation

Kendall's τ rank correlation is a nonparametric measure of association and is calculated from the number of concordant and discordant pairs from two independent copies of a random variable. For example, for two independent copies $W_{i}$, $W_{i^{\prime }}$ of a random vector W, the population‐level Kendall's τ is defined as

$$\tau_{\mathrm{jk}}=E\left[\mathrm{sgn}\left\{\left(W_{\mathrm{ij}}-W_{i^{\prime }j}\right)\left(W_{\mathrm{ik}}-W_{i^{\prime }k}\right)\right\}\right]$$

In the context of functional data, we can define the population Kendall's τ between *j*th and *j*′th time = points. Let us denote $\delta_{jj^{\prime }}^{ii^{\prime }}=\mathrm{sgn}\left\{\left(X_{i}\left(t_{j}\right)-X_{i^{\prime }}\left(t_{j}\right)\right)\left(X_{i}\left(t_{j^{\prime }}\right)-X_{i^{\prime }}\left(t_{j^{\prime }}\right)\right)\right\}$. Then, the population Kendall's τ can be defined as $\tau_{jj^{\prime }}=E\left(\delta_{jj^{\prime }}^{ii^{\prime }}\right)=E\left(\mathrm{sgn}\left\{\left(X_{i}\left(t_{j}\right)-X_{i^{\prime }}\left(t_{j}\right)\right)\left(X_{i}\left(t_{j^{\prime }}\right)-X_{i^{\prime }}\left(t_{j^{\prime }}\right)\right)\right\}\right)$, where $X_{i}\left(\cdot \right)$ and $X_{i^{\prime }}\left(\cdot \right)$ are two independent realizations of random process X(⋅). The population Kendall's τ of observed process can bridged via a monotone function F to the population level latent correlation both for pairs of the same type of data and their combinations as shown in Yoon, Carroll, and Gaynanova [25] and Dey and Zipunnikov [29], that is, $\tau_{jj^{\prime }}=F\left(C\left(t_{j},t_{j^{\prime }}\right)\right)$. The bridging function F depends on the variable types and the marginal cutoffs (see details in Appendix of Supporting Information). The estimation process calculates the sample Kendall's τ as: ${\hat{\tau }}_{jj^{\prime }}=\frac{2}{n\left(n-1\right)}\sum_{1\leq i<i^{\prime }<n}\mathrm{sgn}\left\{\left(X_{i}\left(t_{j}\right)-X_{i^{\prime }}\left(t_{j}\right)\right)\left(X_{i}\left(t_{j^{\prime }}\right)-X_{i^{\prime }}\left(t_{j^{\prime }}\right)\right)\right\}=\frac{2}{n\left(n-1\right)}\sum_{1\leq i<i^{\prime }<n}\delta_{jj^{\prime }}^{ii^{\prime }}={\bar{\delta }}_{jj^{\prime }}$. Then, the next step is to connect the latent correlation using the known bridging function and estimate Kendall's τ.

#### 2.1.2 Objective Function

The bridging function allows us to express the Kendall's τ in terms of the basis expansions of the covariance function. Hence, we can rewrite: $\delta_{jj^{\prime }}=F\left(C\left(t_{j},t_{j^{\prime }}\right)\right)=F\left(g\left(\sum_{k=1}^{d}\sum_{l=1}^{d}u_{\mathrm{kl}}B_{k}\left(t_{j}\right)B_{l}\left(t_{j^{\prime }}\right)\right)\right)$. We seek to find ***U*** that minimize

(4) $$\begin{array}{ll} \tilde{l} & =\sum_{1\leq i<i^{\prime }<n}\;\sum_{1\leq j<j^{\prime }\leq m}{\left(\delta_{jj^{\prime }}^{ii^{\prime }}-\delta_{jj^{\prime }}\right)}^{2}\\ & =\sum_{1\leq i<i^{\prime }<n}\;\sum_{1\leq j<j^{\prime }\leq m}{\left(\delta_{jj^{\prime }}^{ii^{\prime }}-{\bar{\delta }}_{jj^{\prime }}+{\bar{\delta }}_{jj^{\prime }}-\delta_{jj^{\prime }}\right)}^{2}\\ & =\sum_{1\leq i<i^{\prime }<n}\;\sum_{1\leq j<j^{\prime }\leq m}{\left(\delta_{jj^{\prime }}^{ii^{\prime }}-{\bar{\delta }}_{jj^{\prime }}\right)}^{2}+\sum_{1\leq j<j^{\prime }\leq m}\left(\frac{n}{2}\right){\left({\bar{\delta }}_{jj^{\prime }}-\delta_{jj^{\prime }}\right)}^{2}\\ & =\sum_{1\leq i<i^{\prime }<n}\;\sum_{1\leq j<j^{\prime }\leq m}{\left(\delta_{jj^{\prime }}^{ii^{\prime }}-{\bar{\delta }}_{jj^{\prime }}\right)}^{2}+\left(\frac{n}{2}\right)\\ & \;\sum_{1\leq j<j^{\prime }\leq m}({\hat{\tau }}_{jj^{\prime }}-F{\left(g\left(\sum_{k=1}^{d}\sum_{l=1}^{d}u_{\mathrm{kl}}B_{k}\left(t_{j}\right)B_{l}\left(t_{j^{\prime }}\right))\right)\right)}^{2} \end{array}$$

where, $\tilde{l}=\tilde{l}\left(U\right)$ and $\delta_{jj^{\prime }}=F(C(t_{j},t_{j^{\prime }}))=F(g(\sum_{k=1}^{d}\sum_{l=1}^{d}u_{\mathrm{kl}}B_{k}(t_{j})B_{l}(t_{j^{\prime }})))$. Only the second term of the objective function $\tilde{l}$ in (4) depends on ***U***. Hence, minimizing $\tilde{l}$ is equivalent to minimizing the nonlinear least squares objective function $\sum_{1\leq j<j^{\prime }\leq m}{\left({\hat{\tau }}_{jj^{\prime }}-F\left(g\left(\sum_{k=1}^{d}\sum_{l=1}^{d}u_{\mathrm{kl}}B_{k}\left(t_{j}\right)B_{l}\left(t_{j^{\prime }}\right)\right)\right)\right)}^{2}$ with respect to U. This minimization problem hence translates to fitting a regression model using non‐linear least squares [31] to obtain estimate of the coefficients $u_{\mathrm{kl}}$. In particular, we have,

(5) $$\hat{U}=\underset{U}{\text{argmin}}\;\sum_{1\leq j<j^{\prime }\leq m}{\left({\hat{\tau }}_{jj^{\prime }}-F\left(g\left(\sum_{k=1}^{d}\sum_{l=1}^{d}u_{\mathrm{kl}}B_{k}\left(t_{j}\right)B_{l}\left(t_{j^{\prime }}\right)\right)\right)\right)}^{2}$$

Subsequently, $\hat{C}\left(s,t\right)=g\left(\sum_{k=1}^{d}\sum_{l=1}^{d}{\hat{u}}_{\mathrm{kl}}B_{k}\left(s\right)B_{l}\left(t\right)\right),s\neq t,$ is the estimator of the correlation function ($\hat{C}\left(t,t\right)=1$). The Gauss–Newton algorithm [32] within nls function in R is used to solve this non‐linear optimization problem. Note that the objective function in (5) can be expressed as $S\left(U\right)=\sum_{s=1}^{N}r_{s}{\left(U\right)}^{2}$. The Gauss–Newton algorithm finds the value of U iteratively by minimizing this sum of squares. Starting with an initial estimate $U^{\left(0\right)}$, the update at the (*L* + 1)th stage based on the current estimate $U^{\left(L\right)}$ is given by

$$U^{\left(L+1\right)}=U^{\left(L\right)}-{\left(J_{r}^{T}J_{r}\right)}^{-1}J_{r}^{T}r\left(U^{\left(L\right)}\right)$$

Here $r=\left(r_{1},\ldots ,r_{N}\right)$ and $J_{r}$ denotes the Jacobian matrix, with ${\left(J_{r}\right)}_{\mathrm{sq}}=\frac{\partial r_{s}\left(U^{\left(L\right)}\right)}{\partial U_{q}}$. The number of tensor product basis functions d in either direction is restricted to be less than m, which guarantees a unique solution to the normal equations.

#### 2.1.3 Choice of Tuning Parameter

The number of basis functions d controls the smoothness of the estimated latent correlation function. In this article, we follow a truncated basis approach by restricting the number of B‐spline bases to be small in both directions to incorporate smoothness [1, 33]. Note that in the latent scale $\left(V\left(t_{1}\right),\cdots ,V\left(t_{m}\right)\right)∼N\left(0,{\mathbb{C}}_{m\times m}\right)$, where ${\mathbb{C}}_{m\times m}$ is the correlation function C(⋅,⋅) evaluated on the grid $S=\left\{t_{1},t_{2},\ldots ,t_{m}\right\}$. Hence, to choose the number of basis functions in a data‐driven way, we use the following Bayesian Information criterion (BIC) and choose the value of d, which gives the smallest BIC(d).

(6) $$\begin{array}{l} \mathrm{BIC}\left(d\right)=-2\log\hat{L}+\frac{d\left(d+1\right)}{2}\log\left(n\right)\\ -\;2\log\hat{L}=n\log\left(\hat{\mathbb{C}}\right)+\sum_{i=1}^{n}{\hat{V}}_{i}^{T}{\hat{\mathbb{C}}}^{-1}{\hat{V}}_{i}+\mathrm{nm}\log\left(2\pi \right) \end{array}$$

Here ${\hat{V}}_{i}$ denotes the latent predictions (BLUP) of $V_{i}$ which can be obtained as illustrated later in the Section 2.2 and $\hat{\mathbb{C}}$ is the estimated correlation matrix.

#### 2.1.4 Bridging Functions

In our estimation process, we need to know the bridging function F(⋅) introduced in Equation (5). Analytic expressions for the bridging functions are available in the literature for various scenarios, including continuous and binary variables [20], truncated variables [25], and ordinal variables [29]. Given our focus on the marginal distribution of univariate processes (continuous/truncated/ordinal/binary), it is imperative to ascertain the specific analytic forms of the bridging functions that facilitate the transformation of Kendall's τ to latent correlation for pairs of variables falling into the same category (e.g., continuous–continuous, truncated–truncated). We provide these analytic expressions in Appendix A of the Supporting Information.

#### 2.1.5 Estimation of the Cutoff Parameters

Note that the bridging function F(⋅) depends on the cutoffs for pairs that involve binary, ordinal, and truncated functional data [24, 29]. Hence, we follow a method of moments‐based estimation approach described in Dey and Zipunniko [29] to get point‐wise estimates of the cutoff process Δ(⋅). Observe that the cutoff process is only identifiable up to a monotone transformation [20]. Hence, we can only get estimates of $f_{t}\left(\Delta \left(t\right)\right)$; with a slight abuse of notation, we denote the cutoffs as Δ(t) in the estimating equations. From the observed data, we can estimate the cutoffs through the method of moments as follows:

(7) $$\begin{array}{ll} \text{Binary}:\hat{\Delta }\left(t\right) & =\Phi^{-1}\left(\frac{\sum_{i=1}^{n}I\left(X_{i}\left(t\right)=0\right)}{n}\right)\\ \text{Ordinal}:{\hat{\Delta }}_{k}\left(t\right) & =\Phi^{-1}\left(\frac{\sum_{i=1}^{n}I\left(X_{i}\left(t\right)<=\left(k-1\right)\right)}{n}\right),k=1,\ldots ,l-1\\ \text{Truncated}:\hat{\Delta }\left(t\right) & =\Phi^{-1}\left(\frac{\sum_{i=1}^{n}I\left(X_{i}\left(t\right)=0\right)}{n}\right) \end{array}$$

##### 2.1.5.1 Estimation of the Transformation Functions $f_{t}$

We do not need to know the monotone transformation functions for estimating the covariance parameters, and the transformation functions are not estimable for binary and ordinal values [20, 29]. However, we need to get a point‐wise estimate of the transformation function to get latent predictions at the time points for continuous and truncated variables (for non‐zero observed value). We define

(8) $$\begin{array}{l} {\hat{G}}_{t}\left(x\right)=\frac{1}{n+1}\sum_{i=1}^{n}I\left(X_{i}\left(t\right)\leq x\right),x\in \mathbb{R},\left(\text{for continuous process}\right)\\ {\hat{G}}_{t}\left(x\right)=\frac{1}{n+1}\sum_{i=1}^{n}I\left(X_{i}\left(t\right)\leq x\right),x>0,\left(\text{for truncated process}\right) \end{array}$$

Then, we can use Equation (8) to get the estimator of monotone transformations as follows (based on section 4 of Liu, Lafferty, and Wasserman [23])

(9) $$\begin{array}{l} {\hat{f}}_{t}\left(x\right)=\Phi^{-1}\left({\hat{G}}_{t}\left(x\right)\right),\left(\text{for continuous process}\right)\\ {\hat{f}}_{t}\left(x\right)=\Phi^{-1}\left({\hat{G}}_{t}\left(x\right)\right),\left(\text{for truncated process}\right) \end{array}$$

### 2.2 Extension to Sparse Data

The proposed estimation method can be extended to more general scenarios where each curve $X_{i}\left(\cdot \right)$ is observed sparsely and irregularly at time points $S_{i}=\left\{t_{i1},t_{i2},\ldots ,t_{{\mathrm{im}}_{i}}\right\}$. For example, in our motivating application, the intra‐day mood ratings are reported four times per day, which could be at different time points for different subjects. This gives rise to sparse functional data [4]. Although the individual number of observations $m_{i}$ is small, we consider the scenario where $\cup_{i=1}^{n}\cup_{j=1}^{m_{i}}t_{\mathrm{ij}}$ is dense in [0,1] [34]. Let us denote $\cup_{i=1}^{n}\cup_{j=1}^{m_{i}}t_{\mathrm{ij}}=\left\{t_{1},t_{2},\ldots ,t_{M}\right\}$. In this case we can estimate the sample Kendall's τ as: ${\hat{\tau }}_{jj^{\prime }}={\bar{\delta }}_{jj^{\prime }}=\frac{1}{N_{jj^{\prime }}}\sum_{1\leq i<i^{\prime }<n:n_{jj^{\prime }}^{ii^{\prime }}>0}\mathrm{sgn}\left\{\left(X_{i}\left(t_{j}\right)-X_{i^{\prime }}\left(t_{j}\right)\right)\left(X_{i}\left(t_{j^{\prime }}\right)-X_{i^{\prime }}\left(t_{j^{\prime }}\right)\right)\right\}=\frac{1}{N_{jj^{\prime }}}\sum_{1\leq i<i^{\prime }<n:n_{jj^{\prime }}^{ii^{\prime }}>0}\delta_{jj^{\prime }}^{ii^{\prime }}$. Here $n_{jj^{\prime }}^{ii^{\prime }}=1$ if both $X_{i}\left(\cdot \right),X_{i^{\prime }}\left(\cdot \right)$ are observed at time‐points $t_{j}$ and $t_{j^{\prime }}$, $N_{jj^{\prime }}$ denotes the total number of pairwise complete observation at $t_{j},t_{j^{\prime }}$. We extend the nonlinear least square objective function from (5) to the set of all time‐points $\left\{t_{1},t_{2},\ldots ,t_{M}\right\}$, and define the modified objective function for sparse data as follows:

(10) $$\tilde{l}\left(U\right)\;=\sum_{1\leq j<j^{\prime }\leq M:N_{jj^{\prime }}>c_{0}}{\left({\hat{\tau }}_{jj^{\prime }}-F\left(g\left(\sum_{k=1}^{d}\sum_{l=1}^{d}u_{\mathrm{kl}}B_{k}\left(t_{j}\right)B_{l}\left(t_{j^{\prime }}\right)\right)\right)\right)}^{2}$$

Here, we only consider those time points for which there are a sufficient number of pairwise observations, $c_{0}$, to estimate $\tau_{jj^{\prime }}$ accurately and with less variability. Throughout the article, we set $c_{0}=5$, which has resulted in a satisfactory performance, as demonstrated in our empirical analysis.

### 2.3 Curve Prediction

Suppose the curve $X_{i}\left(\cdot \right)$ is observed at time‐points $\left\{t_{i1},t_{i2},\ldots ,t_{{\mathrm{im}}_{i}}\right\}$ and we want to predict $X_{i}\left(\cdot \right)$ at $\left\{s_{i1},s_{i2},\ldots ,s_{\mathrm{im}}\right\}$. This could be important in predicting the trajectory of a subject with partial information. Let us denote the latent observations corresponding to the observed data by $V_{i}^{O}$ and the new data by $V_{i}^{N}$. On latent scale, $\left(\begin{array}{c} V_{i}^{O}\\ V_{i}^{N} \end{array}\right)∼N\left(\left(\begin{array}{c} 0\\ 0 \end{array}\right),\left(\begin{array}{cc} {\mathbb{C}}^{O,O} & {\mathbb{C}}^{O,N}\\ {\mathbb{C}}^{N,O} & {\mathbb{C}}^{N,N} \end{array}\right)\right)$. The individual components of the covariance matrix are obtained from the estimated covariance matrix using methods from Section 2.1. We have

$$E\left(V_{i}^{N}|V_{i}^{O}\right)={\mathbb{C}}^{N,O}{\left\{{\mathbb{C}}^{O,O}\right\}}^{-1}\left(V_{i}^{O}-0\right)$$

$$\mathrm{Cov}\left(V_{i}^{N}|V_{i}^{O}\right)={\mathbb{C}}^{N,N}-{\mathbb{C}}^{N,O}{\left\{{\mathbb{C}}^{O,O}\right\}}^{-1}{\mathbb{C}}^{O,N}$$

Latent predictions can be obtained as ${{\hat{V}}_{i}}^{N}={\hat{\mathbb{C}}}^{N,O}{\hat{\mathbb{C}}}^{O,O-1}\left({{\hat{V}}_{i}}^{O}-0\right)$, where ${{\hat{V}}_{i}}^{O}=E\left(V_{i}^{0}\left(\cdot \right)|X_{i}^{0}\left(\cdot \right)\right)$ is the best linear unbiased predictor (BLUP) based on the proposal of Dey and Zipunnikov [29]. ${{\hat{X}}_{i}}^{N}$ can be obtained from ${{\hat{V}}_{i}}^{N}$ using the estimated cut‐off parameters and the estimated transformation functions. For example, for binary X(⋅), we would have ${{\hat{X}}_{i}}^{N}\left(t\right)=I\left({{\hat{V}}_{i}}^{N}\left(t\right)>{\hat{\Delta }}_{t}\right)$.

### 2.4 Latent Principal Components and Scores

For continuous functional data $X_{i}\left(t\right)$, we can use truncated Karhunen‐Loeve expansion of $X_{i}\left(\cdot \right)$ as $X_{i}\left(t\right)\approx \mu \left(t\right)+\sum_{k=1}^{K}\zeta_{\mathrm{ik}}\psi_{k}\left(t\right)$, where $\zeta_{\mathrm{ik}}$ are mean zero functional principal component (FPC) scores with variance $\lambda_{k}$ and $\psi_{k}\left(t\right)$ are orthogonal eigenfunctions [4]. Let ∑(s,t) denote the covariance function of X(⋅), that is, ∑(s,t)=Cov(X(s),X(t)). By Mercer's theorem, the covariance kernel has the following spectral decomposition $\sum \left(s,t\right)=\sum_{k=1}^{\infty }\lambda_{k}\psi_{k}\left(s\right)\psi_{k}\left(t\right)$. The eigenvalues and eigenfunctions hence can be estimated from the eigenequation $\int_{0}^{1}\sum \left(s,t\right)\phi_{k}\left(t\right)\mathrm{dt}=\lambda_{k}\psi_{k}\left(t\right)$. This procedure is referred to as FPCA. Uncorrelated scores $\zeta_{\mathrm{ik}}$ serve as a multivariate summary of infinite‐dimensional data and are widely used in many applications. For truncated or discrete functional data X(t), the KL expansion is not directly applicable. We can instead use the estimated latent correlation function $\hat{C}\left(s,t\right)$ to perform FPCA. We use the eigenequation $\int_{0}^{1}\hat{C}\left(s,t\right)\psi_{k}^{L}\left(t\right)\mathrm{dt}=\lambda_{k}\psi_{k}^{L}\left(t\right)$, to estimate the latent eigenfunctions, which reveals the major modes of variations in the data. The scores of the latent representations V(t) can be obtained using standard FPCA techniques [4], if V(t) was available. To obtain latent FPC scores, we use the estimated smoothed covariance function $\hat{C}\left(s,t\right)$ to obtain BLUPs of latent representations $\hat{V}\left(\cdot \right)=E\left(V\left(\cdot \right)|X\left(\cdot \right)\right)$ [29] and use FPCA on latent representations $\hat{V}\left(t\right)$ to estimate latent FPC scores $\zeta_{\mathrm{ik}}^{L}$.

## 3 Simulation Study

In this section, we investigate the performance of the proposed estimation method via numerical simulations. To this end, the following scenarios are considered.

### 3.1 Data Generating Scenarios

In simulations, we consider four data types—binary, ordinal, truncated, and continuous. For each data type, the latent functional process V(⋅) is generated from either of the following two correlation functions.

#### 3.1.1 Stationary Covariance $C_{1}\left(\cdot ,\cdot \right)$

In this case $V\left(\cdot \right)∼𝒢𝒫\left(0,C_{1}\left(\cdot \cdot \right)\right)$, where 𝒢𝒫(μ(⋅),C(⋅⋅)) denotes a Gaussian process with mean function μ(⋅) and covariance function C(⋅,⋅). Here, we take $C_{1}\left(s,t\right)$ to be the Matern correlation kernel with parameters $\sigma^{2}=1$, ν=3.5 and $\tau =\frac{1}{7}$. The covariance kernel of a general Matérn process with parameters $\nu ,\sigma^{2},\tau$ is given by $C_{1}\left(s,t\right)=\sigma^{2}\frac{2^{1-\nu }}{\Gamma \left(\nu \right)}{\left(\sqrt{2\nu }\frac{d}{\tau }\right)}^{\nu }K_{\nu }\left(\sqrt{2\nu }\frac{d}{\tau }\right),\;d=|s-t|$, where $K_{\nu }$ is the modified Bessel function of the second kind.

#### 3.1.2 Non‐Stationary Covariance $C_{2}\left(\cdot ,\cdot \right)$

In this case, the latent process V(t) is generated again as $V\left(\cdot \right)∼𝒢𝒫\left(0,C_{2}\left(\cdot \cdot \right)\right)$, where $C_{2}\left(\cdot ,\cdot \right)$ is a non‐stationary correlation function given by $C_{2}\left(s,t\right)=\frac{C_{2}^{*}\left(s,t\right)}{\sqrt{C_{2}^{*}\left(s,s\right)}\sqrt{C_{2}^{*}\left(t,t\right)}}$ and the covariance function $C_{2}^{*}\left(s,t\right)=0.05^{2}2\sin\left(\pi t\right)\sin\left(\pi s\right)+0.09^{2}2\cos\left(\pi t\right)\cos\left(\pi s\right)+0.01^{2}I\left(s=t\right)$.

#### 3.1.3 Scenario A: Binary Functional Data

In this case, the observed functional data X(t) is generated from the latent functional data V(t) as $X\left(t\right)=I\left(V\left(t\right)>\Delta_{t}\right)$, where $\Delta_{t}=0.5$ is used for all t. We consider a dense and equispaced grid of m=50 time‐points S in 𝒯=[0,1] for the observed data X(t). Two sets of correlation function $C_{1}\left(\cdot ,\cdot \right)$, and three sets of sample size n∈{100,500,1000} are considered for data generation.

#### 3.1.4 Scenario B: Ordinal Functional Data

In this case, the observed functional data X(t) is generated as,

$$X\left(t\right)=\left\{\begin{array}{l} 0\;\text{if}-\infty \leq V\left(t\right)<-0.6\\ 1\;\text{if}-0.6\leq V\left(t\right)<0.1\\ 2\;\text{if}\;0.1\leq V\left(t\right)<0.6\\ 3\;\text{if}\;0.6\leq V\left(t\right)<\infty \end{array}\right.$$

The rest of the simulation design (dense grid), including covariance kernels and sample sizes, are kept exactly the same as in scenario A.

#### 3.1.5 Scenario C: Truncated Functional Data

The observed functional data X(t) is generated based on the latent process V(⋅) as,

$$X\left(t\right)=\left\{\begin{array}{l} 0\;\text{if}\;V\left(t\right)<0.5\\ V\left(t\right)\;\text{if}\;0.5\leq V\left(t\right) \end{array}\right.$$

The rest of the simulation design is again kept the same as in scenario A (dense).

#### 3.1.6 Scenario D: Continuous Functional Data

The observed functional data X(t) is generated as a continuous transformation of latent process V(⋅) as,

$$X\left(t\right)={\left\{V\left(t\right)\right\}}^{3}$$

The rest of the simulation design is kept the same as in scenario A (dense). We also consider an additional simulation scenario D2, for continuous functional data and sample size n=1000, where X(t)=V(t), and the rest of the design is kept the same.

#### 3.1.7 Additional Scenario E: Sparse Functional Data

Another simulation scenario with n=1000 considers all four (binary, ordinal, truncated, and continuous) functional data types generated according to a sparse design. After generating $X_{i}\left(t\right)$ as in scenarios A‐D, we assume that each curve $X_{i}\left(t\right)$ is observed sparsely at a set of randomly chosen 40% time‐points from S. For the stationary covariance function, particularly for this sparse scenario, we use a Matern correlation kernel with parameters $\sigma^{2}=1$, ν=3.5 and $\tau =\frac{1}{2}$. The non‐stationary covariance kernel is kept the same.

### 3.2 Simulation Results

We generated 100 Monte‐Carlo (MC) replications from each of the simulation scenarios to assess the performance of the proposed estimation method. In all the scenarios, we use seven cubic B‐spline basis functions (over both arguments) to model the latent correlation function C(s,t) using tensor product splines over 𝒯×𝒯. We compare our proposed approach (denoted henceforth as FSGC) to a few alternatives: (i) naive FPCA using observed curves X(t), which captures the within‐curve correlation in the observed data, (ii) binary FPCA (BFPCA) proposed by Wrobel et al. [15], (iii) correlation estimated using huge.npn function within the huge package [21] in R, which assumes the data to be continuous while estimating latent correlation using SGC [20] (we expect our results to be aligned with this method in scenario D when the data type is continuous), and (iv) we also use FPCA on the latent predictions from multivariate (non‐functional) SGCRM [29] (referred henceforth as FSGC Latent). Note that FSGC Latent is essentially a naive version of our proposed method treating functional observations as multivariate and smoothing is performed in the last step. This could be inefficient, for example, in sparse and irregular designs. We compare the performance of FSGC with other competing methods in terms of the integrated square errors for estimation of the covariance functions, where ISE is defined as $\mathrm{ISE}=\int_{0}^{1}\int_{0}^{1}{\left\{C_{T}\left(s,t\right)-\hat{C}\left(s,t\right)\right\}}^{2}\text{dsdt}$, where $C_{T}\left(s,t\right)$ is the true covariance function and $\hat{C}\left(s,t\right)$ is the estimated covariance. The ISE values of the estimators across several data‐types (Scenario A–D) and covariance kernel are reported in Table 1 for sample size n=500. Similar metrics for sample size n=100,n=1000 are reported in Tables S1 and S2.

**TABLE 1:** Average ISE $\times 10^{3}$ (and corresponding SD) between true and estimated covariance matrices for different methods and scenarios (n=500).

	Stationary				Non‐stationary			
Scenario	FSGC	FSGC Latent	BFPCA	Huge.npn	FSGC	FSGC Latent	BFPCA	Huge.npn
A (Binary)	**2 (1)**	4 (2)	23 (9)	23 (5)	**2 (1)**	**2 (1)**	6 (1)	51 (4)
B (Ordinal)	**1 (1)**	2 (1)	NA	3 (1)	**1 (1)**	**1 (1)**	NA	3 (1)
C (Truncated)	**2 (2)**	3 (2)	NA	11 (4)	2 (1)	**1 (1)**	NA	44 (4)
D (Continuous)	**1 (1)**	**1 (1)**	NA	**1** (1)	0.9 (0.8)	**0.7 (0.7)**	NA	0.8 (0.8)

*Note*: The minimum average ISE is presented in bold‐face.

#### 3.2.1 Performance Under Scenario A

We compare the performance of FSGC with BFPCA, huge.npn and FSGC Latent in terms of the integrated square errors (ISE) for estimation of the covariance functions. The results from naive FPCA are not numerically comparable with FSGC as it captures the correlation structure in the observed data, hence we only report the estimated covariance surfaces which give us an idea of what the estimated covariance surface would be if we treated the observed data as continuous functional data.

**FIGURE 3:**
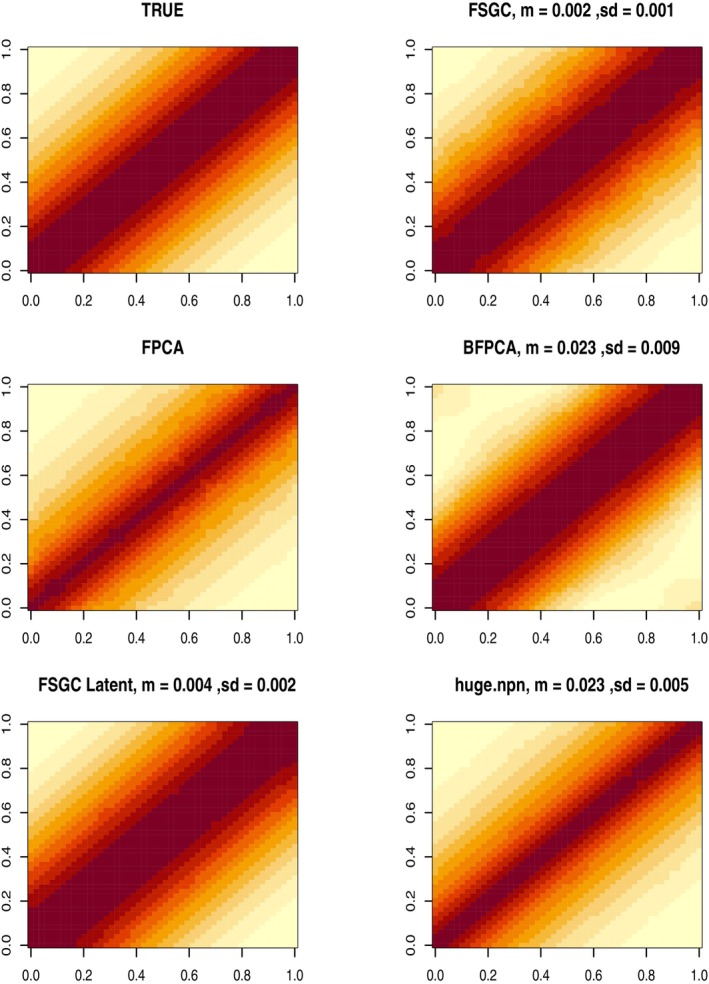
True and estimated average covariance surface for stationary covariance kernel, scenario A, *n* = 500. Average ISE (and SD) of the estimates are reported on the top of each image. FSGC denotes the proposed FSGC method, FPCA denotes naive FPCA of the observed data, BFPCA denotes binary FPCA, FSGC Latent denotes FPCA on latent predictions from SGCRM. We also display the covariance obtained using huge.npn function.

Figure 3 displays the average estimated covariance surface over the grid 𝒯×𝒯 from the five estimation approaches along with the true covariance surface for the stationary covariance case and n=500. The mean (*m*) and standard deviation (SD) of ISE are reported for all cases using MC replications in Table 1. We observe that the proposed FSGC method clearly outperforms binary FPCA in terms of estimation accuracy based on the average ISE. In particular, the proposed FSGC approach is seen to produce 10 times smaller ISE compared to BFPCA and huge.npn. The average ISE from the FSGC approach is also found to be two times smaller than the FSGC Latent approach, highlighting the importance of smoothing.

Figure S1 displays the average estimated covariance surface and the estimates for the non‐stationary covariance case and n=500. We observe that the proposed FSGC method again produces superior performance compared to the binary FPCA and huge.npn method. The average ISE from the direct FSGC approach is found to be comparable to the FSGC Latent approach in this case. The results for n=100,1000 are similar for stationary and non‐stationary scenarios, where a superior performance of the FSGC method can be noted and are reported in Tables S1 and S2.

We apply the proposed FSGC method to estimate latent scores as outlined in Section 2.3. The FSGC latent (multivariate) approach is also used. Note that the latent curves are identifiable only up to a monotone transformation. In Table S4, we report the correlation between the estimated latent scores and the estimated scores (using FPCA) from the true latent curves. We observe that the first two scores are strongly correlated with their estimated counterparts across all sample sizes and covariance types. For this particular scenario and sample size n=500, we also report the average integrated square errors (ISE) between the true and the estimated eigenfunctions in Table S5. The estimation performance of the eigenvalues is also illustrated in Figures S11 and S12. Both of these results highlight the impressive performance of the FSGC method compared to its competitors in capturing the principal components.

We also investigated the sensitivity of the proposed estimation method to the number of basis functions used for modeling, which plays the role of tuning parameters. This is reported in Table S6 for a varying number of basis functions for sample size n=500. In particular, the performance of the proposed method is noticed to be not too sensitive to the value of this tuning parameter as long as a truncated basis approach is used by restricting the number of B‐spline bases to be small in both directions to incorporate smoothness [1, 33]. The performance from the BIC chosen tuning parameter can be noted to be similar to the number of basis functions used (7) for this scenario. We report a comparison of computation time of the proposed FSGC, FSGC latent approach with the BFPCA approach, for sample size n=500 in Table S7. A satisfactory performance of the proposed approach can be observed, taking into consideration the performance of the methods, although the computation time can be noted to increase with increasing basis dimensions.

#### 3.2.2 Performance Under Scenario B

**FIGURE 4:**
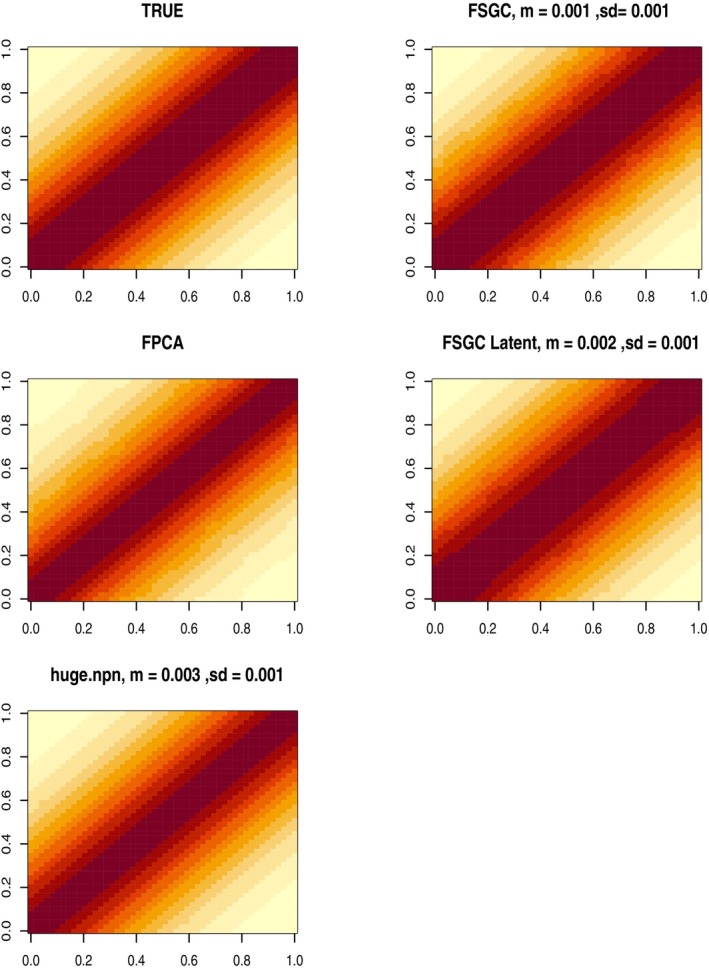
True and Estimated average covariance surface for stationary covariance kernel, scenario B, *n* = 500. Average ISE (and SD) of the estimates are reported on the top of the respective images. FSGC denotes the proposed estimation method, FPCA denotes FPCA on the observed curve and FSGC Latent is FPCA on latent predictions from SGCRM. We also display the covariance obtained using huge.npn function.

We apply (i) the proposed FSGC method along with (ii) FPCA on the observed curve X(t) and (iii) FSGC Latent method (iv) huge.npn method to estimate the latent correlation function C(s,t). The performance of the estimators is reported in terms of the integrated square errors (except naive FPCA) (ISE) in Table 1 for n=500 and in Tables S1 and S2 for the other sample sizes. Figure 4 displays the average estimated correlation surface over the grid 𝒯×𝒯 from the four estimation approaches, along with the true surface for the stationary covariance kernel and sample size n=500.

It can be seen that under the ordinal scenario, the proposed FSGC (and FSGC Latent) approach clearly outperforms the marginal huge.npn approach producing almost three times smaller ISE. The results are similar for the other stationary and non‐stationary covariance scenarios across the sample sizes, where a superior performance of the proposed FSGC method can be noted. Figure S2 displays the estimated covariance matrices for the non‐stationary covariance case and n=500.

#### 3.2.3 Performance Under Scenario C, D

The performance of the proposed estimation method under scenarios C, and D are reported in Table 1 for n=500 and Tables S1 and S2 for sample sizes n=100,1000. Figures S3–S6 display the estimated covariance matrices for scenario C and D for sample size n=500, and for the stationary and non‐stationary covariance cases respectively. We observe the FSGC approach to produce 10–40 times smaller ISE compared to huge.npn for the truncated scenario. For the continuous scenario D, we observe a similar performance across all the competing methods, which is expected. Figures S7 and S8 present the estimated covariance matrices for additional scenario D2 for sample size n=1000, and for the stationary and non‐stationary covariance cases. We observe that directly applying FPCA yields marginally better performance in this case, which is expected as X(t) itself is continuous and Gaussian functional data.

#### 3.2.4 Performance Under Sparse Scenario E

The results from the additional simulation scenario E (sparse design) along with the performance of the curve prediction method for Binary functional data are presented in Appendix B of Supporting Information, where a superior performance of the FSGC is evident.

Overall, the proposed FSGC method can be observed to estimate the true covariance function more accurately across all four data types and covariance scenarios. Thus, it provides a unified framework for covariance estimation for continuous, truncated, and discrete functional data.

## 4 Application to mHealth NIMH Family Study

We apply our approach to mHealth data collected in the National Institute of Mental Health Family Study of the Mood Spectrum Disorders [35, 36]. The study is a large community‐based study with participants recruited from a community screening of the greater Washington, DC, metropolitan area. Our analysis focuses on a sample of 497 participants, ranging in age from 7 to 84. Table S4 reports other demographics of the sample.

Participants used a smartphone app to rate their current mood on a Likert scale from (1) to (7) (very happy to very sad, with (4) = neutral) four times per day during 7 a.m.–11 p.m. time period each day for fourteen consecutive days. Ratings (5), (6), and (7) were collapsed into a single group for this analysis due to a very small number of participants reporting in those ranges, resulting in 5 ordinal categories for emotional states. Our key research interest is to better understand within‐day temporal patterns of reported mood states and to determine whether differences in these patterns are associated with affective disorders. For our analysis, we focus on the midpoints of sixteen 1‐h windows (m=16) starting at 7 a.m. and ending at 11 p.m. (the windows are 7–8 a.m., 8–9 a.m., ⋯, 10 –11 p.m. with corresponding midpoints at 7:30 a.m., 8:30 a.m., etc.). We consider N=6591 subject‐day‐level functional curves of ordinal type across all subjects to estimate the latent correlation and the eigenfunctions. In principle, this resembles the marginal FPCA approach in Park and Staicu [37] for estimating the marginal covariance and the marginal (intra‐day) eigenfunctions, which is of the main interest in this paper. An extension of the proposed approach to multi‐level or longitudinal functional data will be pursued in future extensions.

**FIGURE 5:**
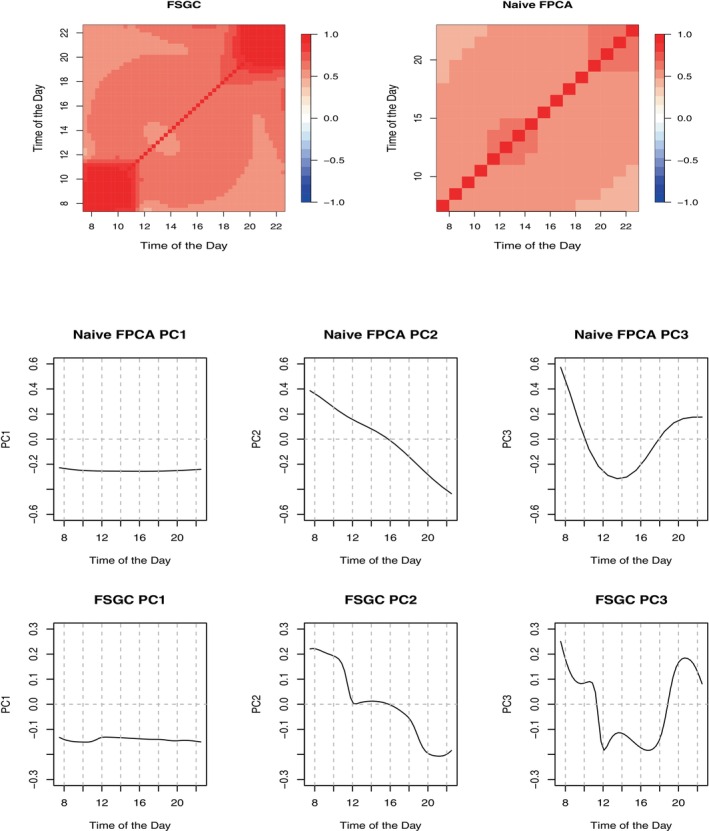
Top panel shows estimated FSGC (left) and naive FPCA (right) correlation matrices. The middle and bottom panels show the first three naive FPCA and FSGC FPCs, respectively.

Several pairs of time‐combinations have a small number of observations introducing a large amount of missingness (≈80%) and sparsity. In this case, due to the large amount of sparsity, the competing huge.npn function [21] fails to estimate the latent correlation matrix. We apply the proposed FSGC method from Section 2.1.5.1 for sparse functional data, which is able to handle this situation. We use K=6 cubic B‐spline basis functions with knots equally spaced between 7:30 a.m. and 10:30 p.m. to model the latent covariance. The number of basis functions was chosen using the proposed BIC in Section 2.1.5.1. Our analysis proceeds as follows. First, we obtain and interpret estimated latent covariance and principal components. Second, we calculate predictions of latent representations of the observed data and use them to obtain latent principal component scores. Finally, we aggregate subject‐specific scores into subject‐specific mean and standard deviations of scores and associate those summaries with affective disorder diagnosis. The top panel of Figure 5 displays the estimated FSGC and naive FPCA estimated covariances.

Note that the naive FPCA captures the covariance between observed data, which is shown here for illustrative purposes. Visually compared to naive FPCA, FSGC covariance demonstrates larger and more complex within‐day temporal dependence, and morning to early noon FSGC correlations (8 a.m.–12 p.m.) are higher. The bottom panel of Figure 5 shows the first three FSGC FPCs that capture approximately 66%,10%, and 4% of total variability, respectively. The middle panel of Figure 5 shows the first three naive FPCs that capture approximately 57%,6%, and 3% of total variability, respectively. Note that both approaches provide visually similar estimated FPCs. This is expected as the number of ordinal categories is relatively large, and we have a very large sample size. FSGC FPCs can be interpreted as follows. Interpreting scientifically, FPC1 captures a temporally global daily mean of happiness/unhappiness; FPC2 estimates a contrast between the morning part of the day (earlier than 12 p.m.) and the evening part (6 p.m. and later); FPC3 captures the degree of evening return to the morning happiness/unhappiness level. Hence, subjects with lower FPC1 scores will be happier than subjects with higher scores; subjects with higher (positive) FPC2 scores will be less happy in the morning compared to the late evening. Finally, subjects with higher (positive) FPC3 scores will have a more pronounced “return” to the morning levels. It is interesting to note that FPC3 from naive FPCA has an evening return level significantly lower than the morning level. In contrast, FPC3 from FSGC has very similar morning and evening levels. A bimodal diurnal pattern in the FPC3 from FSGC can also be observed.

**FIGURE 6:**
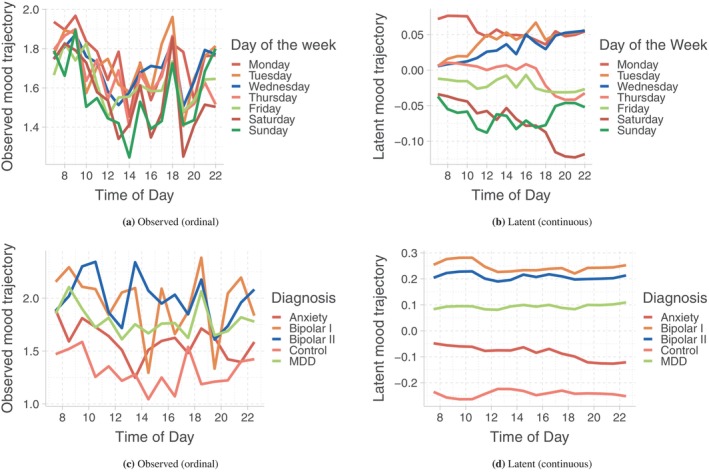
Top panel shows within‐day temporal patterns of mood averaged within each day of the week for observed ratings (left) and their latent representations (right). Bottom panel shows within‐day temporal patterns of mood averaged within each mood disorder subtypes for observed ratings (left) and their latent representations (right).

Another interesting application of our approach is estimating latent representations of reported mood and understanding time‐of‐day and day‐of‐week differences in their temporal patterns. We compare day‐of‐week average latent temporal patterns in the top panel of Figure 6. Our goal is to learn the effect of social schedules on emotional states. The latent trajectory reveals a clear clustering in the night‐time levels of happiness: lowest in the early week (Monday to Wednesday), increases in the midweek (Thursday to Friday), and reaches highest in the weekend (Saturday to Sunday). This separation in the diurnal pattern of emotional states between days of the week is not visually apparent in the observed trajectories.

Next, we explore the within‐day temporal patterns of mood disorder subtypes. Latent trajectories averaged within each diagnosis (Figure 6 bottom panel), reveal an ordering of unhappiness between the diseases: Bipolar I > Bipolar II > MDD > Anxiety > Control. Within‐day temporal trajectories are flatter than observed ones, but there is a much clearer separation between diagnosis groups. We calculate the latent subject‐day mood scores and aggregate them using average and standard deviation for each subject across days. This gives us three mean and three SD latent FPC scores for each subject. Figure S13's top and bottom panels display the distribution of the latent mean FPC scores and SD FPC scores, respectively, for five mood‐disorder groups. It appears that bipolar I, bipolar II, and MDD groups have lower mean FPC‐1 scores compared to the controls. Anxiety and MDD groups seem to have higher SD FPC‐1 scores than controls. Similarly, Bipolar II is observed to have a higher SD FPC‐2 score compared to controls. Figure S14 displays the correlation between mean and SD FPC scores. SD FPC scores are moderately positively correlated. We fit a multinomial logistic regression model for mood disorders subtypes with controls being the reference and mean and SD FPC scores as predictors while adjusting for age and sex (with male being the reference). Since SD FPC scores are correlated, we include them one at a time along with the mean FPC scores in competing models and choose the one with the lowest AIC. Table 2 displays the estimated regression coefficients along with their associated *p*‐values (Wald test) for the chosen multinomial logistic regression model of affective disorders on the mean of FPC1, FPC2, FPC3, and SD of FPC2 scores. We use α=0.05 for the statistical significance of all the tests. A higher mean FPC1 score is found to be associated with lower odds of bipolar I, bipolar II disorders, and MDD relative to controls. This clearly demonstrates that these diagnosis groups report different levels of daily mood. A higher SD FPC2 score, capturing the variability of the FPC2 score across days, is found to be associated with higher odds of bipolar II and MDD disorders relative to controls. Note that FPC2 was illustrated to capture a contrast between the morning part of the day (earlier than 12 p.m.) and the evening part (6 p.m. and later). Hence, the across‐day variability of SD FPC2 score would reflect an across‐day variability in the contrast between morning and evening mood for this person, which could be attributed to occupation, weekend–weekday, and other factors.

**TABLE 2:** Estimated regression coefficients from multinomial logistic regression of mood disorders (Type) on the mean of the first three FSGC FPCs and the SD of FSGC FPC‐2 scores, age, and sex.

Type	Intercept	Age	Sex (female)	Mean FPC‐1	Mean FPC‐2	Mean FPC‐3	SD FPC‐2
Anxiety	−0.04 (0.940)	−0.024 (0.001)	0.84 (0.003)	−0.31 (0.186)	−2.74 (0.113)	−5.44 (0.195)	1.21 (0.501)
Bipolar I	−1.27 (0.050)	−0.0002 (0.981)	0.88 (0.010)	−1.38 (< 0.001)	−2.44 (0.236)	1.01 (0.843)	−0.72 (0.758)
Bipolar II	−2.00 (0.003)	−0.013 (0.140)	0.55 (0.112)	−1.19 (< 0.001)	−0.44 (0.829)	−5.99 (0.223)	6.29 (0.004)
MDD	−1.41 (0.004)	0.003 (0.664)	1.10 (< 0.001)	−0.91 (< 0.001)	−0.47 (0.763)	−3.21 (0.396)	4.06 (0.015)

*Note: p*‐values are reported in the parenthesis.

The proposed FSGC method provides novel insights into the within‐day temporal patterns of mood and their association with different mood disorder subtypes.

## 5 Discussion

The proposed covariance estimation is built on a tensor product spline representation of the covariance of the latent process. The number of basis functions (B‐splines) controls the smoothness and the variance of the covariance estimated with a truncated basis [1, 33]. Our approach is to choose it using a BIC. Alternatively, a roughness penalty can be incorporated in the objective function [1, 5, 6]. This would result in a penalized nonlinear least square problem. We leave the latter approach and choice of smoothing parameter selection as a direction to be explored more deeply in the future. Our empirical analysis has illustrated that the variance of the estimators is not too sensitive to it. In this article, we have followed a point‐wise estimation strategy for cutoff Δ(t) and transformation $f_{t}$ functions (for continuous and truncated data). Since no explicit smoothness is enforced, this formulation allows for very flexible models, including rough, non‐continuous observed processes X(t). If desired, smoothness can be enforced in Δ(t) and $f_{t}\left(\cdot \right)$ by first estimating it point‐wise and, then, smoothing via splines or kernels. The estimated cutoff parameters Δ(t) used in the analytical expression of the bridging function F(⋅) are an additional source of uncertainty in the covariance estimates.

Multiple research directions remain to be explored based on this current work. FSGC method can be used to define distances between two non‐Gaussian, truncated, or discrete functional curves (of the same type) $X_{i}\left(\cdot \right),X_{j}\left(\cdot \right)$ based on the distances between latent trajectories $V_{i}\left(\cdot \right),V_{j}\left(\cdot \right)$, which is the same as the Euclidean distance between estimated latent scores ${\left\{\zeta_{\mathrm{ik}}^{L},\zeta_{\mathrm{jk}}^{L}\right\}}_{k=1}^{K}$ as

(11) $$d\left(X_{i}\left(\cdot \right),X_{j}\left(\cdot \right)\right)={\left\|\zeta_{i}^{L}-\zeta_{j}^{L}\right\|}_{2}$$

**FIGURE 7:**
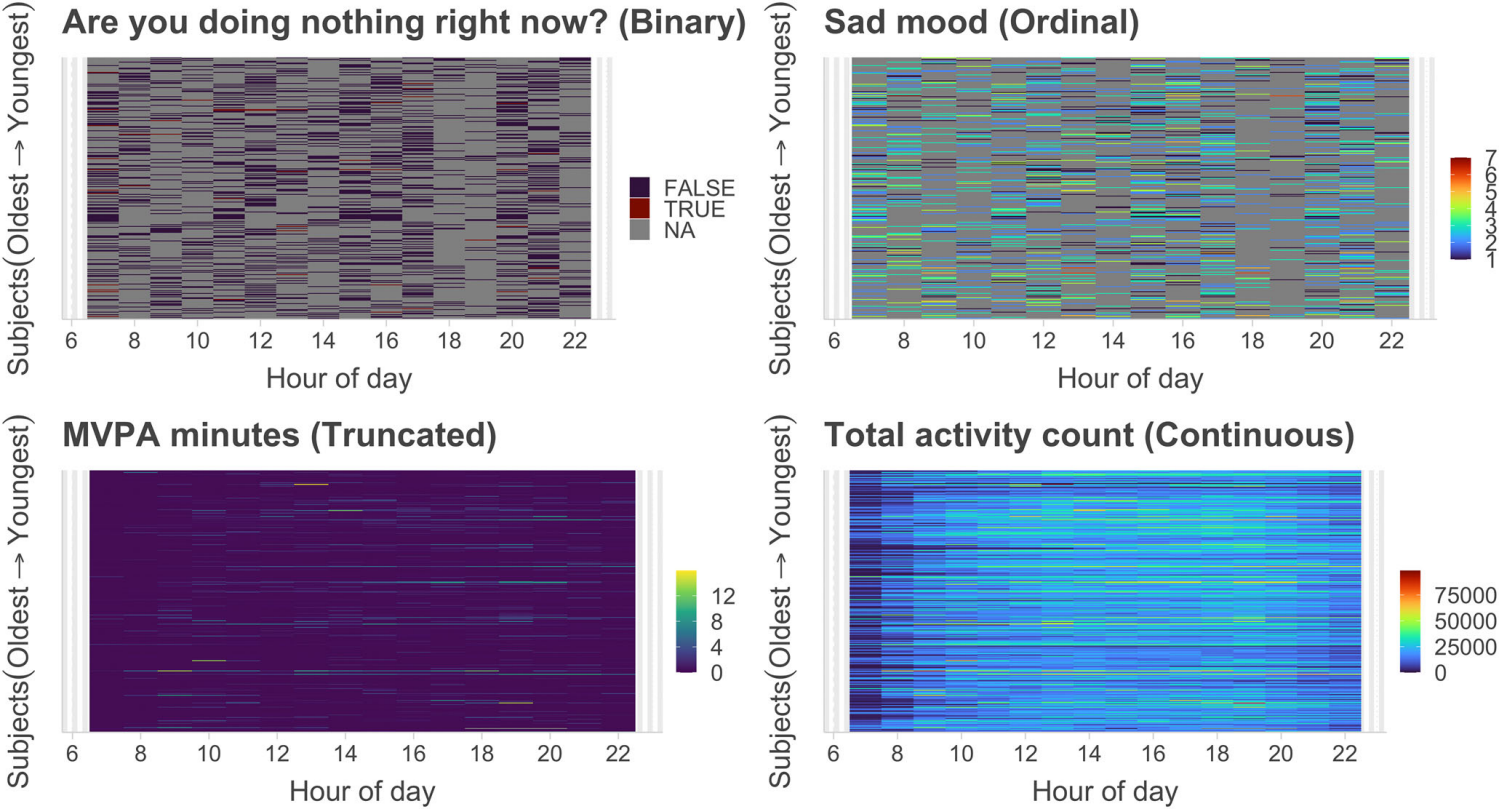
Lasagna plot demonstrating four data‐types (binary, ordinal, truncated, and continuous) observed in the mobile digital health data from NIMH Family study. The grey color denotes missing values. The data presented here is aggregated across subjects and days. It illustrates diurnal variability from 6 a.m. to 10 p.m. MVPA minutes denotes the number of minutes the subject experienced moderate‐to‐vigorous physical activity (≥2200 counts/min) during that hour.

This metric captures the distance between latent trajectories $V_{i}\left(\cdot \right),V_{j}\left(\cdot \right)$ due to the orthogonality of the latent eigenfunctions. This provides a novel way to extend distance‐based clustering approaches [38] to non‐Gaussian functional data. We have focused on modeling univariate functional data of the same type in this article. Alternatively, modeling multivariate mixed‐type functional data would be an important next step that would model the joint latent dependence between different mixed types of data [29] such as diurnal patterns of physical activity (continuous), MVPA minutes (truncated), self‐reported questions (binary), and mood (ordinal). Figure 7 illustrates the need for such analysis in mobile health studies, where the observed data is of mixed‐type. This step is crucial since building separate marginal models and ignoring the cross‐correlation between different variables leads to information loss that might result in biased predictions [16]. Since the proposed FSGC approach is for single‐level functional data, one of the limitations of our application was ignoring the day‐level correlation within the same subject. Following existing methods for multilevel and longitudinal FPCA [37, 39, 40], future extensions of FSGC will be developed to consider multi‐level or longitudinal designs such as the original design of the mHealth data considered in this paper. Additionally, FSGC is primarily developed for moderately dense and sparse designs. The method proposed in this article would not directly scale well to situations like ultra‐dense functional data, for example, densely collected minute‐level physical activity and heart rate data (1440 observations per day) for thousands or tens of thousands of samples. The computational complexity of the proposed method is majorly driven by the calculation of sample Kendall's Tau $\left(O\left(n\log n\left(\frac{m}{2}\right)\right)\right)$ and optimizing the non‐linear least squares objective function. In future work, the recently proposed fast covariance estimation approaches (e.g., FACE) for single‐level and multi‐level continuous functional data [5, 6, 40] could possibly be adopted to FSGC or the FSGC latent approach, which will be able to handle the above high dimensional scenarios. The approach can similarly be extended to highly sparse and irregular functional data using kernel‐weighted objective functions that borrow information from neighboring observations. Extending the proposed method to such a general class of models would allow more diverse applications and remain areas for future research based on this current work.

## 6 Software

An R package implementation of the FSGC estimation has been made available as fpca.sgc.lat function as part of the SGCTools package (https://github.com/Ddey07/SGCTools). We also present a reproducible RMarkdown demonstrating the use of the package in Github and a synthetic data analysis (https://github.com/Ddey07/SGCTools/tree/master/Synthetic_data_analysis) mimicking the data analysis presented in the paper.

## Conflicts of Interest

The authors declare no conflicts of interest.

## Supporting information


Data S1.

## Data Availability

The data that support the findings of this study are available from National Institute of Mental Health. Restrictions apply to the availability of these data, which were used under license for this study. Data are available from the author(s) with the permission of National Institute of Mental Health.
