# Supplementary material for: Functional Principal Component Analysis for Continuous Non‐Gaussian, Truncated, and Discrete Functional Data
Source: Stat Med. 2024 Oct 23;43(28):5431–45. doi: 10.1002/sim.10240 (PMC11586909; doi:10.1002/sim.10240)
Supplement: Supplementary file 1 — Data S1. [file SIM-43-5431-s001.pdf]

Supplementary Material for Functional Principal Component Analysis for Continuous non-Gaussian, Truncated, and Discrete Functional Data

1 Appendix A: Analytic forms of bridging functions

Analytic expressions for the bridging functions are available in the literature for various scenarios, including continuous and binary variables (Liu et al., 2012), truncated variables (Yoon et al., 2018), and ordinal variables (Dey and Zipunnikov, 2022). Given our focus on the marginal distribution of univariate mixed processes (continuous/truncated/ordinal/binary), it is imperative to ascertain the specific analytic forms of the bridging functions that facilitate the transformation of Kendall's τ to latent correlation for pairs of variables falling into the same category (e.g., continuous-continuous, truncated-truncated). We provide these analytic expressions below.

We denote $\rho_{jj'} = C(t_j, t_{j'})$ and the suffices of F - {cc, tt, oo, bb} denote specific cases of continuous, truncated, ordinal and binary variables respectively.

$$
\begin{aligned}
F_{\text{cc}}(\rho_{jj'}) &= \frac{2}{\pi}\sin^{-1}(\rho_{jj'}) \\
F_{\text{bb}}(\rho_{jj'}; \Delta(t_j), \Delta(t'_j)) &= 2\left\{\Phi_2(\Delta(t_j), \Delta(t_{j'}); \rho_{jj'}) - \Phi(\Delta(t_j))\Phi(\Delta(t_{j'}))\right\} \\
F_{\text{tt}}(\rho_{jj'}; \Delta(t_j), \Delta(t_{j'})) &= -2\Phi_4(-\Delta(t_j), -\Delta(t_{j'}), 0, 0; S_{4a}(\rho_{jj'})) + 2\Phi_4(-\Delta(t_j), -\Delta(t_{j'}), 0, 0; S_{4b}(\rho_{jj'})) \\
F_{\text{oo}}(\rho_{jj'}; \Delta(t_j), \Delta(t_{j'})) &= 2\sum_{r=1}^{l_j-1}\sum_{s=1}^{l_{j'}-1}[\Phi_2(\Delta_r(t_j), \Delta_s(t_{j'}); \rho_{jj'})\{\Phi_2(\Delta_{(r+1)}(t_j), \Delta_{(s+1)}(t_{j'}); \rho_{jj'}) - \\
&\quad \Phi_2(\Delta_{(r+1)}(t_j), \Delta_{(s-1)}(t_{j'}; \rho_{jj'})\}] - 2\sum_{r=1}^{l_j-1}\Phi(\Delta_r(t_j))\Phi_2(\Delta_{(r+1)}(t_j), \Delta_{(l_{j'}-1)(t_{j'})}; \rho_{jj'})
\end{aligned}
\tag{S1}
$$

with

$$
S_{4b}(\rho_{jj'}) = \begin{pmatrix} 1 & \rho_{jj'} & 1/\sqrt{2} & \rho_{jj'}/\sqrt{2} \\ \rho_{jj'} & 1 & \rho_{jj'}/\sqrt{2} & 1/\sqrt{2} \\ 1/\sqrt{2} & \rho_{jj'}/\sqrt{2} & 1 & \rho_{jj'} \\ \rho_{jj'}/\sqrt{2} & 1/\sqrt{2} & \rho_{jj'} & 1 \end{pmatrix}
$$

2 Appendix B: Additional Simulation Scenario E Results

Additional simulation under scenario E:

The estimation performance of the proposed FSGC and FSGC latent method for this sparse design and for all 4 types, i.e., binary, ordinal, truncated and continuous functional data are reported in Supplementary Table S3. In this sparse case, the competing methods, e.g., bfpca and `huge.npn` method is no longer applicable. Based on the average ISE, the proposed FSGC method can be observed to provide a robust and satisfactory performance in all these sparse scenarios under different data types.

The estimated correlation surfaces for sparse binary functional data under stationary and non-stationary covariance cases are reported in Figure S9-S10. We observe that the proposed FSGC method outperforms the FSGC latent approach in this case, highlighting the applicability of the proposed approach in sparse designs.

For the sparse and binary functional data scenario, we also apply the proposed curve prediction method in Section 2.3 of the paper and obtain $\hat{X}_i(t)$ at all time points over $\mathcal{S}$ (a dense grid on $\mathcal{T}$). We calculate the accuracy of these binary predictions by comparing them with the true curves $X_i(t)$ across all time points and all subjects. While calculating accuracy, we ignore the points S_i, where the curve $X_i(\cdot)$ was already observed. For a particular M.C replication this is calculated as $ACC = \frac{1}{n}\sum_{i=1}^{n}\frac{1}{m-n_i}\sum_{t_j\in\mathcal{S}-S_i} I(X_i(t_j) = \hat{X}_i(t_j))$. The average accuracy across all M.C replications is found to be 0.98 (sd = 0.002) and 0.95 (sd = 0.002) for the stationary and non-stationary covariance scenario, respectively, illustrating the satisfactory performance of the proposed method in predicting the binary curves at new-time points.

3 Supplementary Figures

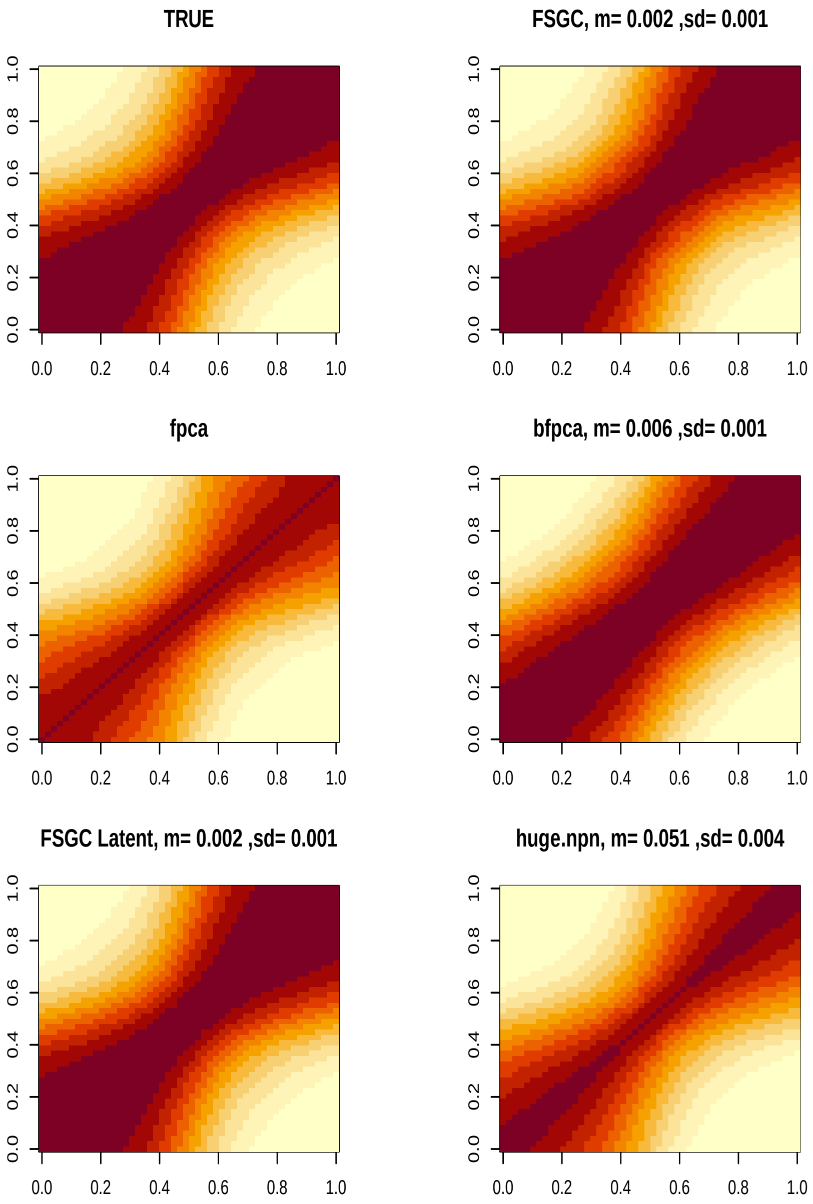

Figure S1: True and estimated average covariance surface for non-stationary covariance kernel, scenario A, n=500. The average ISE (and sd) of the estimates are reported on the top of each image. FSGC denotes the proposed FSGC method, fpca denotes naive FPCA of the observed data, bfpca denotes binary FPCA, and FSGC Latent denotes FPCA on latent predictions from SGCRM. Also displayed is the covariance obtained using `huge.npn` function.

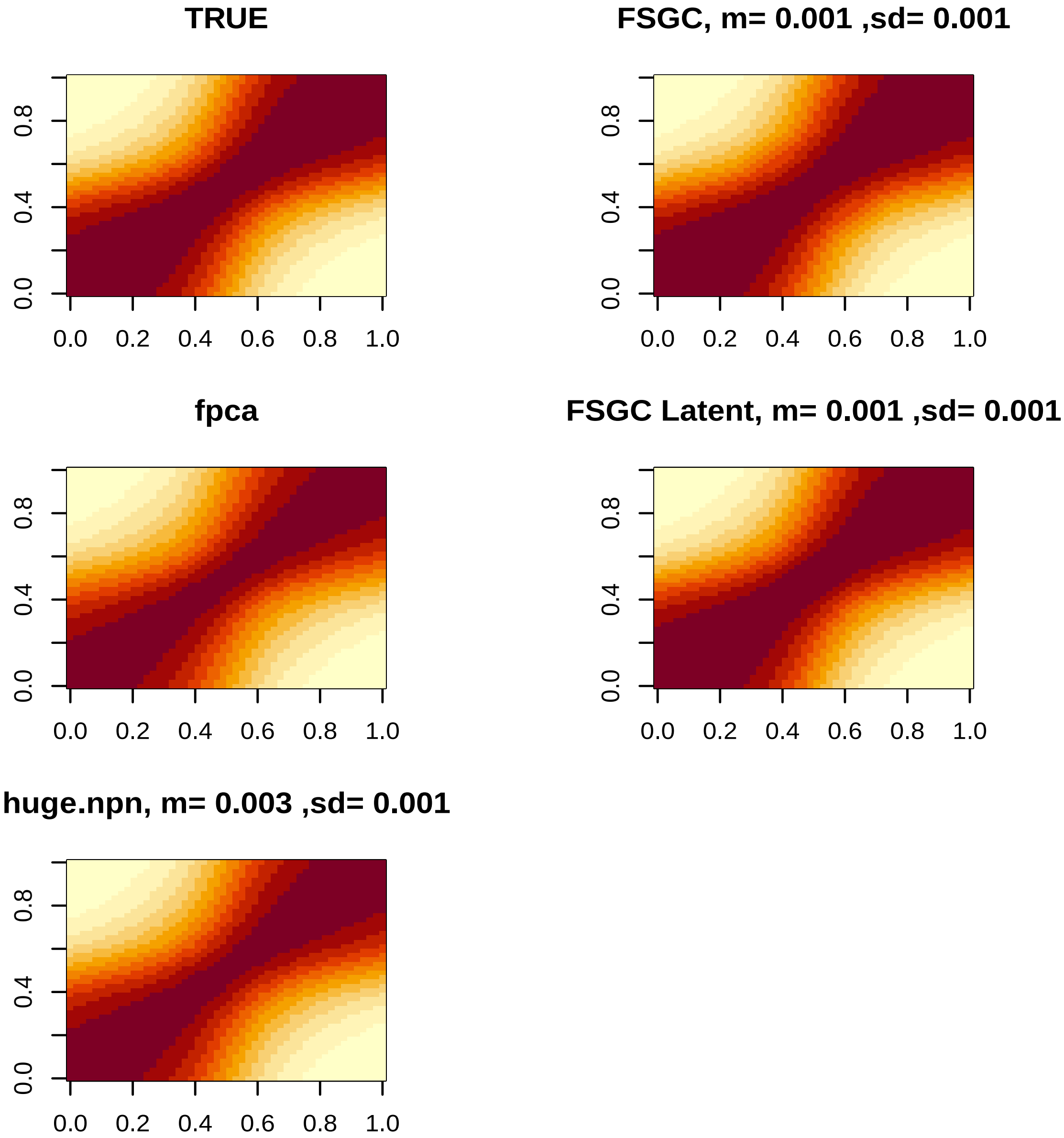

Figure S2: True and Estimated average covariance surface for non-stationary covariance kernel, scenario B, n=500. Average ISE (and sd) of the estimates are reported on the top of the respective images. FSGC denotes the proposed estimation method, fpca denotes FPCA on the observed curve and FSGC Latent is FPCA on latent predictions from SGCRM. Also displayed is the covariance obtained using `huge.npn` function.

Truncated Functional Data

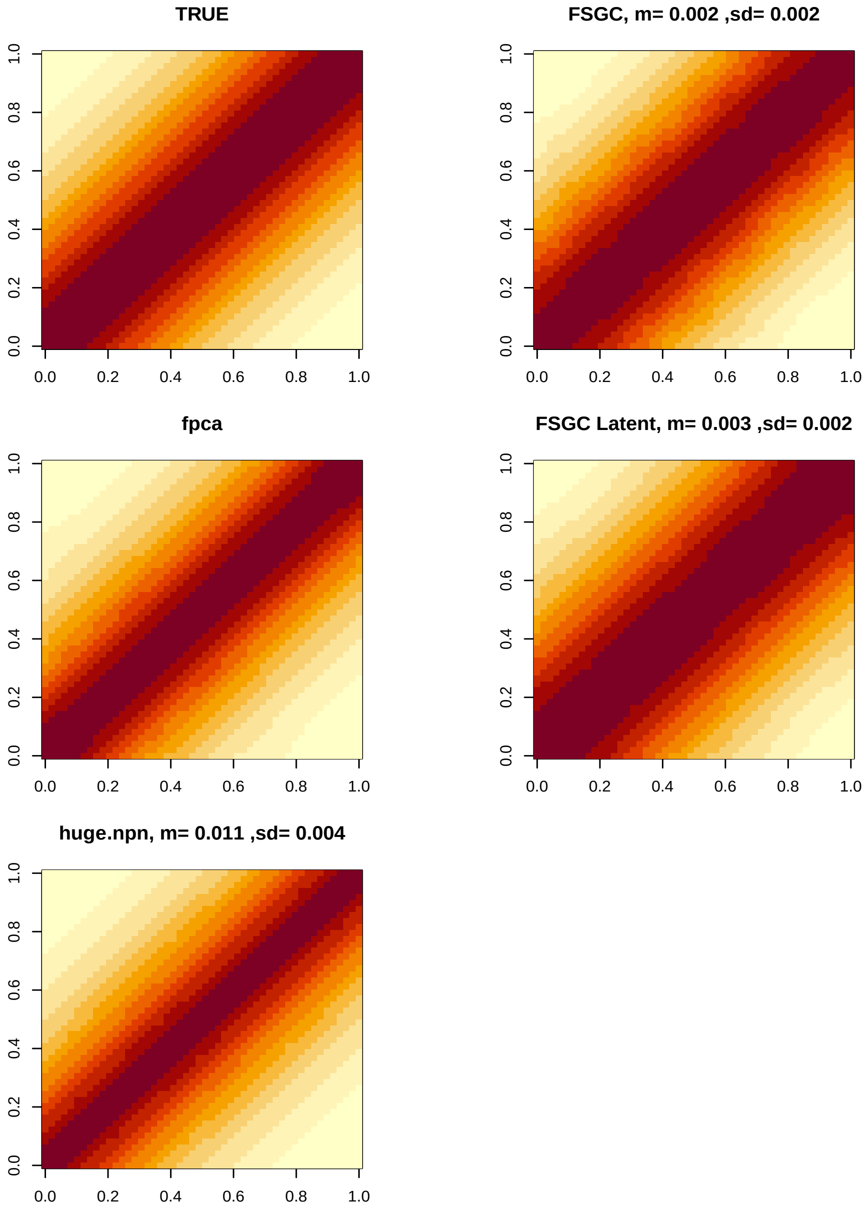

Figure S3: True and Estimated average covariance surface for stationary covariance kernel, scenario C, n=500. Average ISE (and sd) of the estimates are reported on the top of the respective images. FSGC denotes the proposed estimation method, fpca denotes FPCA on the observed curve and FSGC Latent is FPCA on latent predictions from SGCRM. Also displayed is the covariance obtained using `huge.npn` function.

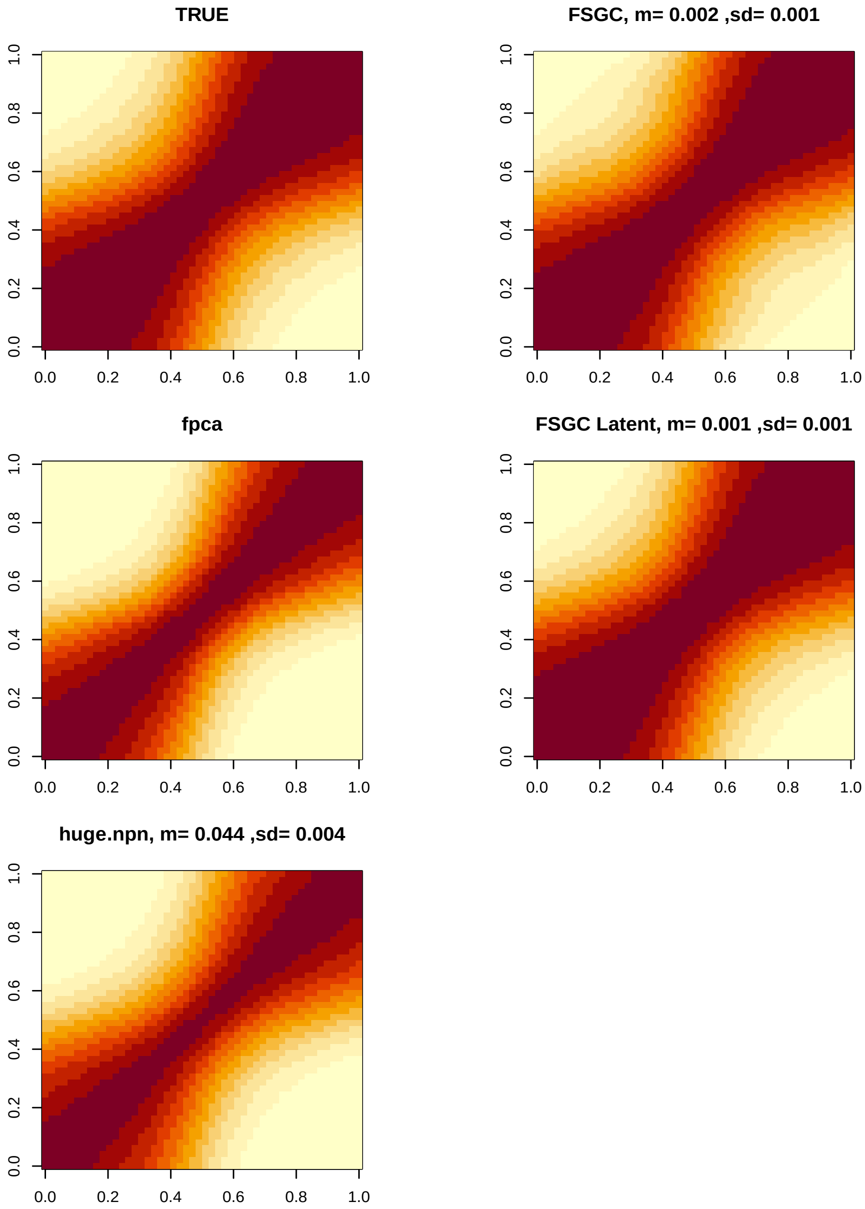

Figure S4: True and Estimated average covariance surface for non-stationary covariance kernel, scenario C, n=500. Average ISE (and sd) of the estimates are reported on the top of the respective images. FSGC denotes the proposed estimation method, fpca denotes FPCA on the observed curve and FSGC Latent is FPCA on latent predictions from SGCRM. Also displayed is the covariance obtained using `huge.npn` function.

Continuous Functional Data

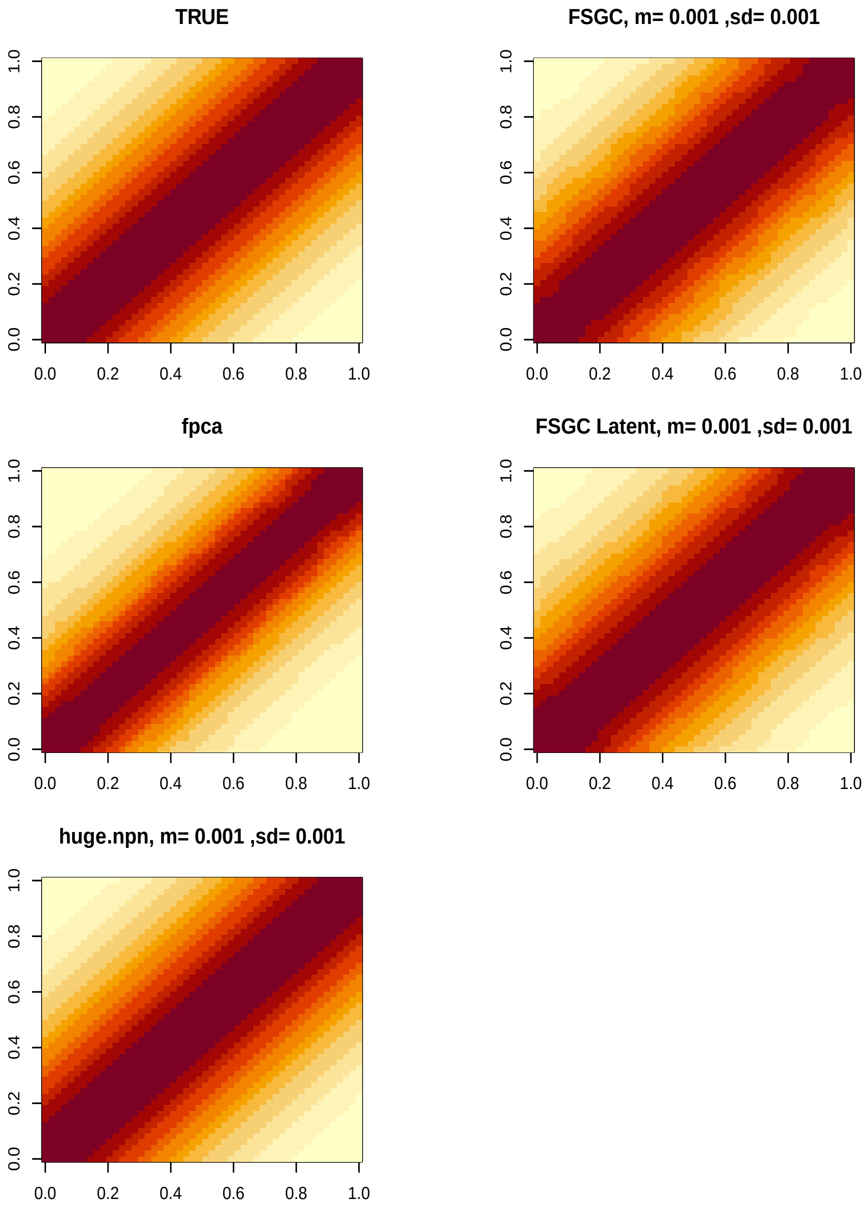

Figure S5: True and Estimated average covariance surface for stationary covariance kernel, scenario D, n=500. Average ISE (and sd) of the estimates are reported on the top of the respective images. FSGC denotes the proposed estimation method, fpca denotes FPCA on the observed curve and FSGC Latent is FPCA on latent predictions from SGCRM. Also displayed is the covariance obtained using `huge.npn` function.

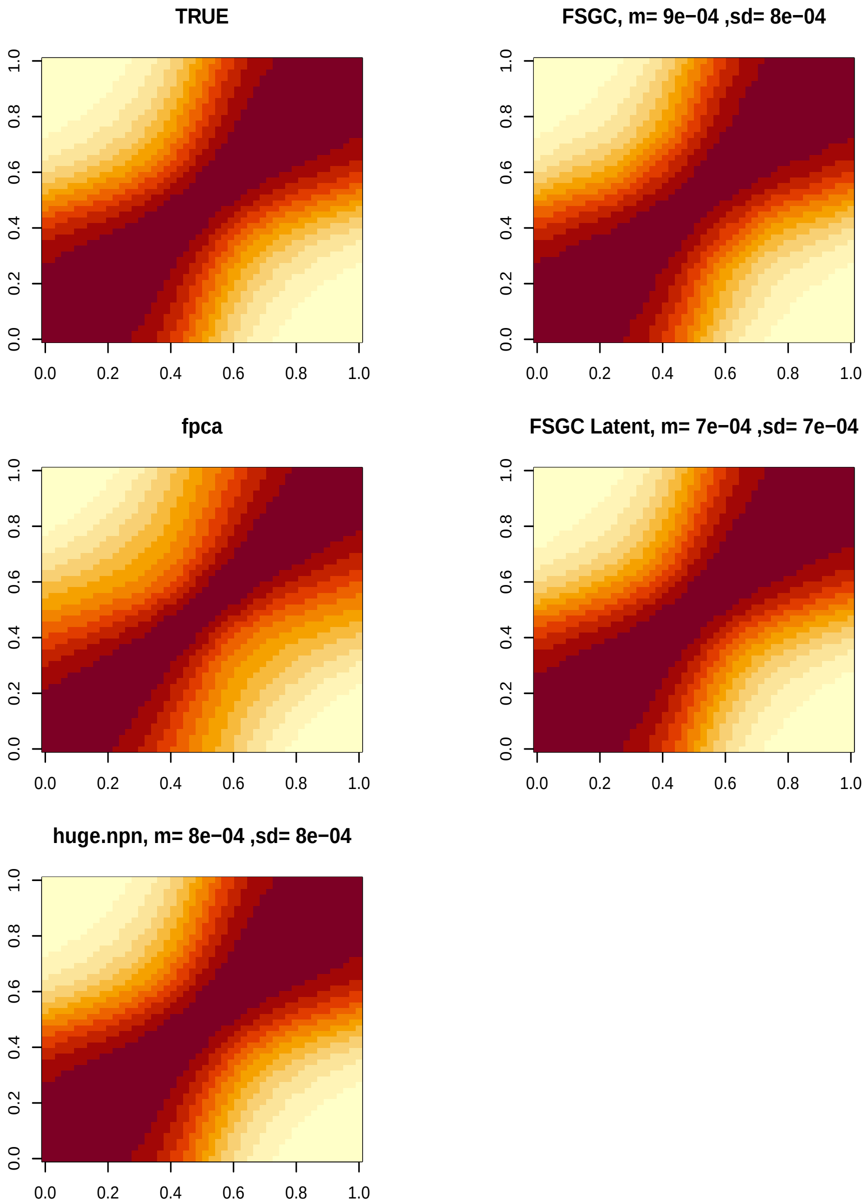

Figure S6: True and Estimated average covariance surface for non-stationary covariance kernel, scenario D, n=500. Average ISE (and sd) of the estimates are reported on the top of the respective images. FSGC denotes the proposed estimation method, fpca denotes FPCA on the observed curve and FSGC Latent is FPCA on latent predictions from SGCRM. Also displayed is the covariance obtained using `huge.npn` function.

Continuous Functional Data: Additional Scenario D2

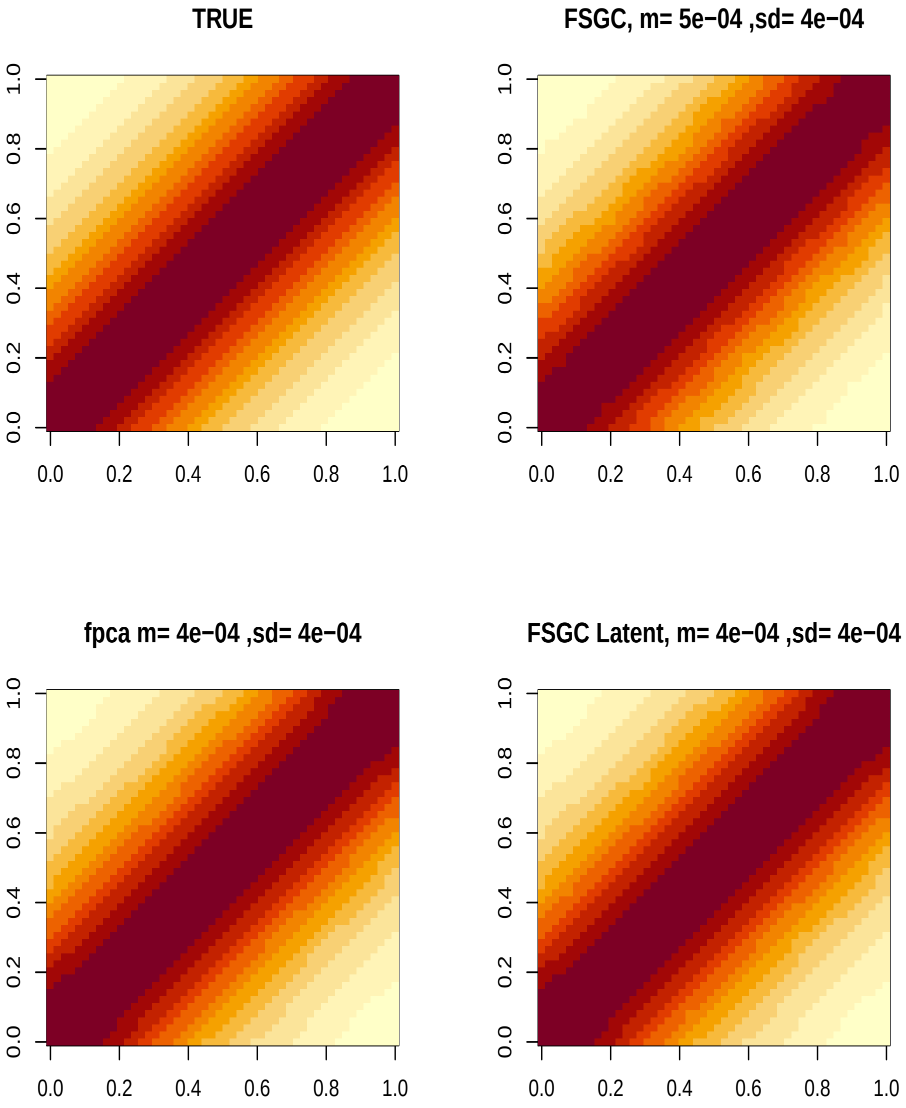

Figure S7: True and Estimated average covariance surface for stationary covariance kernel, scenario D2, n=1000. Average ISE (and sd) of the estimates are reported on the top of the respective images. FSGC denotes the proposed estimation method, fpca denotes FPCA on the observed curve and FSGC Latent is FPCA on latent predictions from SGCRM.

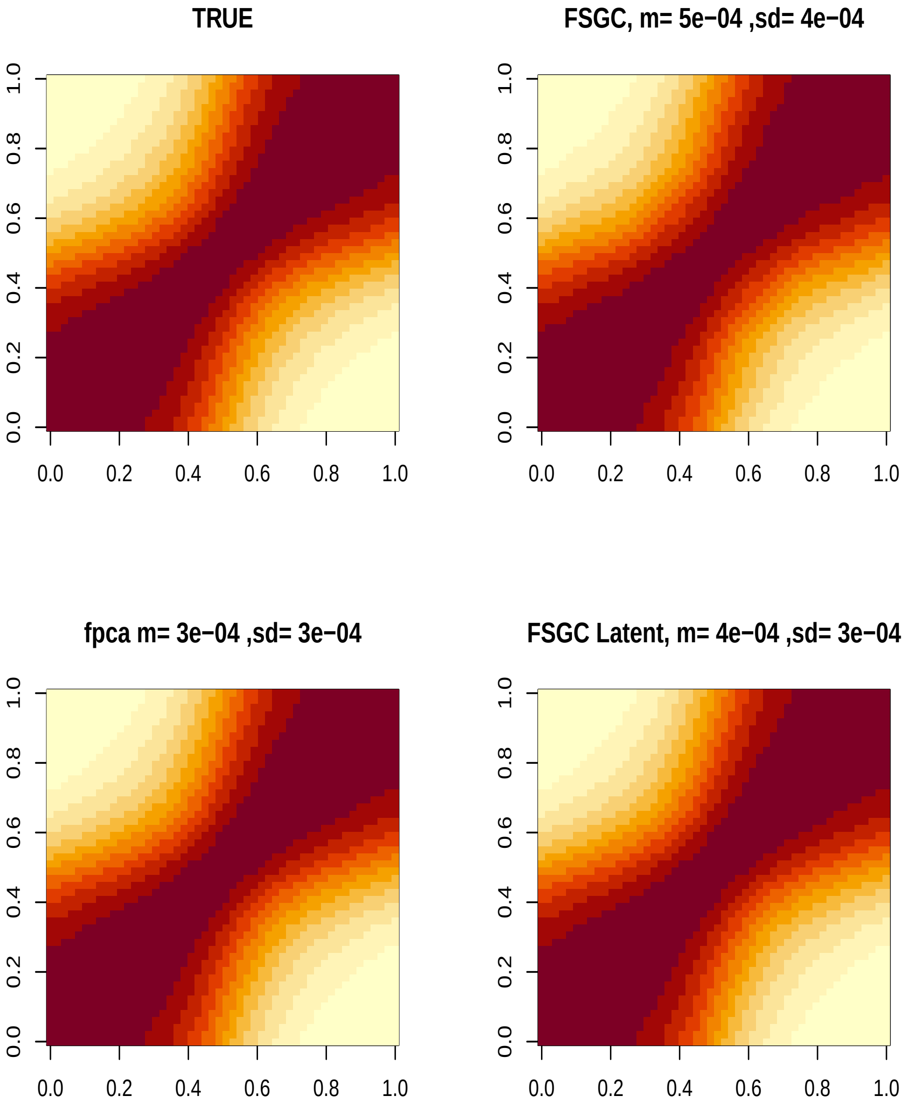

Figure S8: True and Estimated average covariance surface for non-stationary covariance kernel, scenario D2, n=1000. Average ISE (and sd) of the estimates are reported on the top of the respective images. FSGC denotes the proposed estimation method, fpca denotes FPCA on the observed curve and FSGC Latent is FPCA on latent predictions from SGCRM.

Scenario E, Sparse design, Binary Functional Data

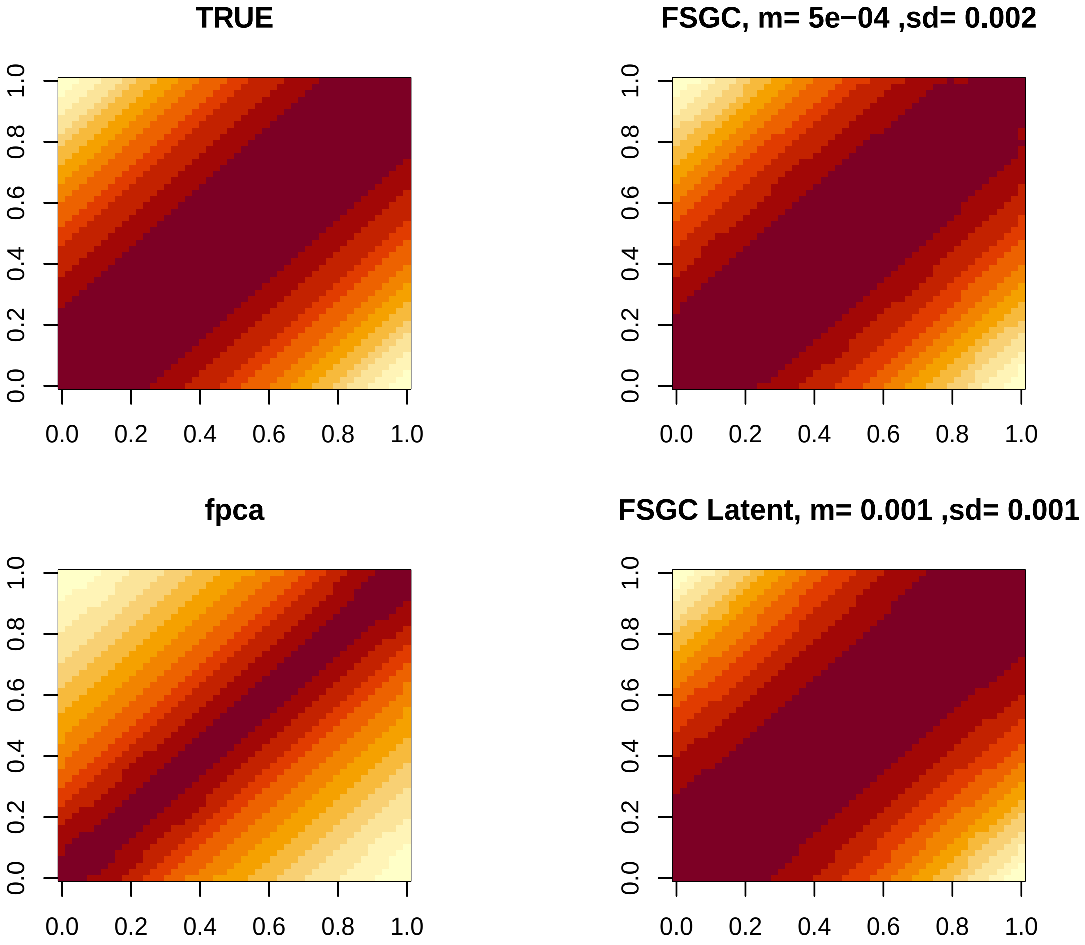

Figure S9: True and Estimated average covariance surface for stationary covariance kernel, sparse design, scenario A, n=1000. Average ISE (and sd) of the estimates are reported on the top of the respective images. FSGC denotes the proposed estimation method, fpca denotes FPCA on the observed curve and FSGC Latent is FPCA on latent predictions from SGCRM.

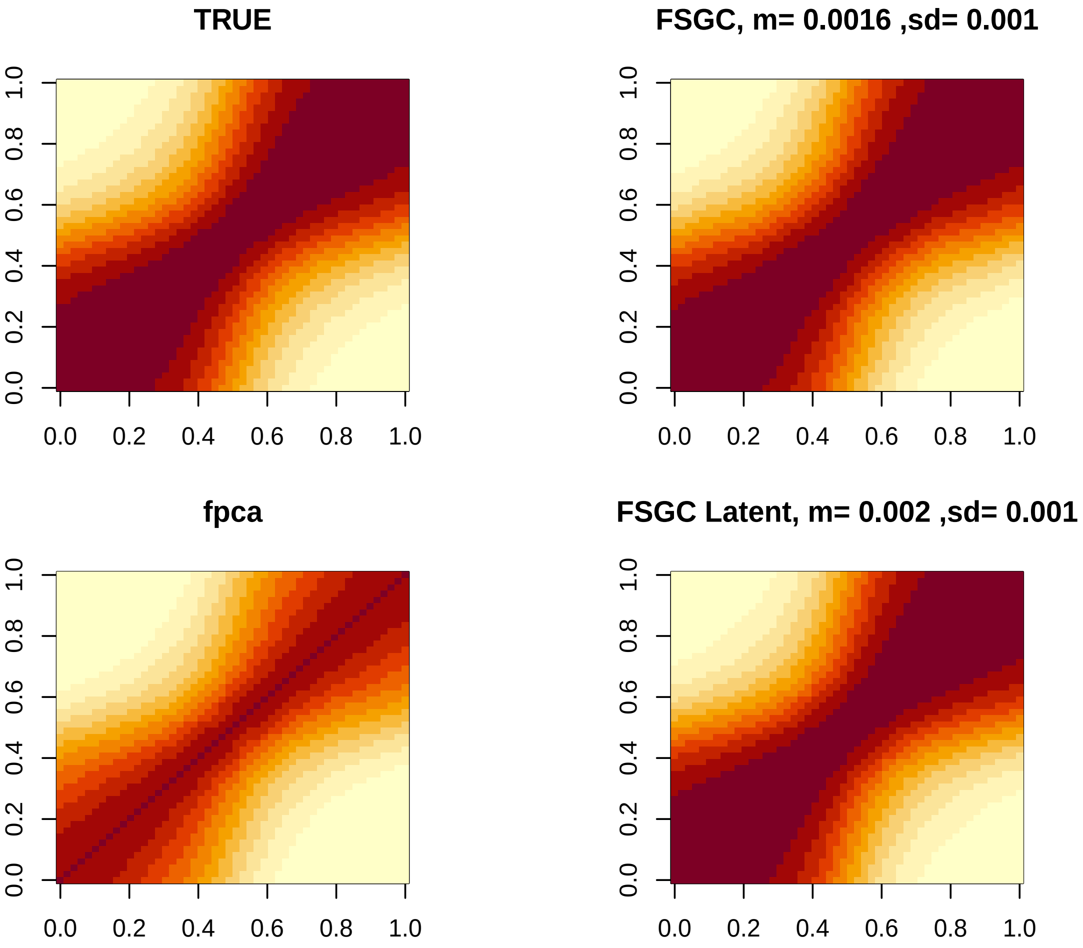

Figure S10: True and Estimated average covariance surface for non-stationary covariance kernel, sparse design, scenario A, n=1000. Average ISE (and sd) of the estimates are reported on the top of the respective images. FSGC denotes the proposed estimation method, fpca denotes FPCA on the observed curve and FSGC Latent is FPCA on latent predictions from SGCRM.

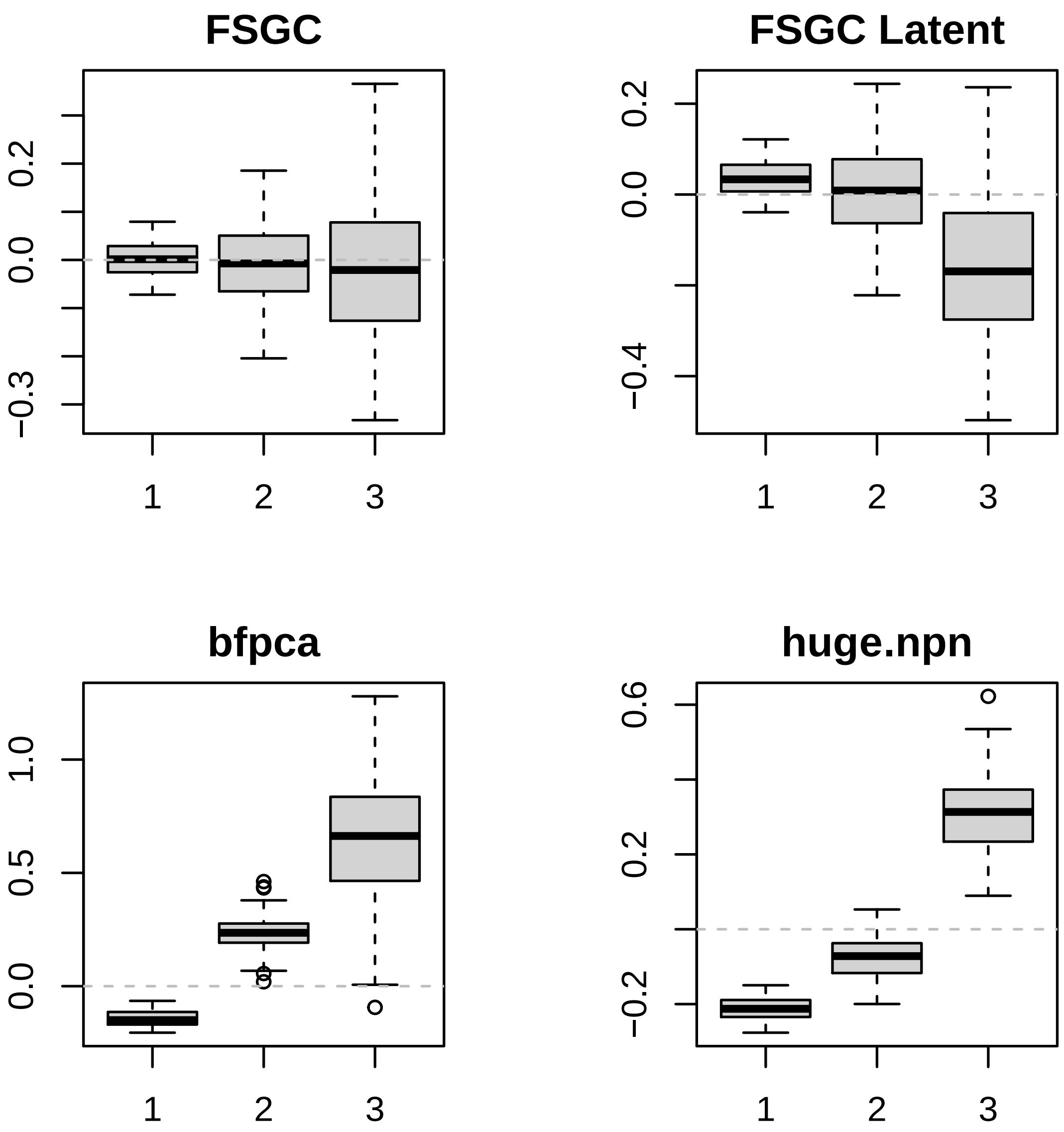

Figure S11: Displayed are the distribution of estimated eigenvalues divided by true eigenvalue −1 ($\frac{\hat{\lambda}_k}{\lambda_k} - 1$), for scenario A, n=500 and stationary covariance function.

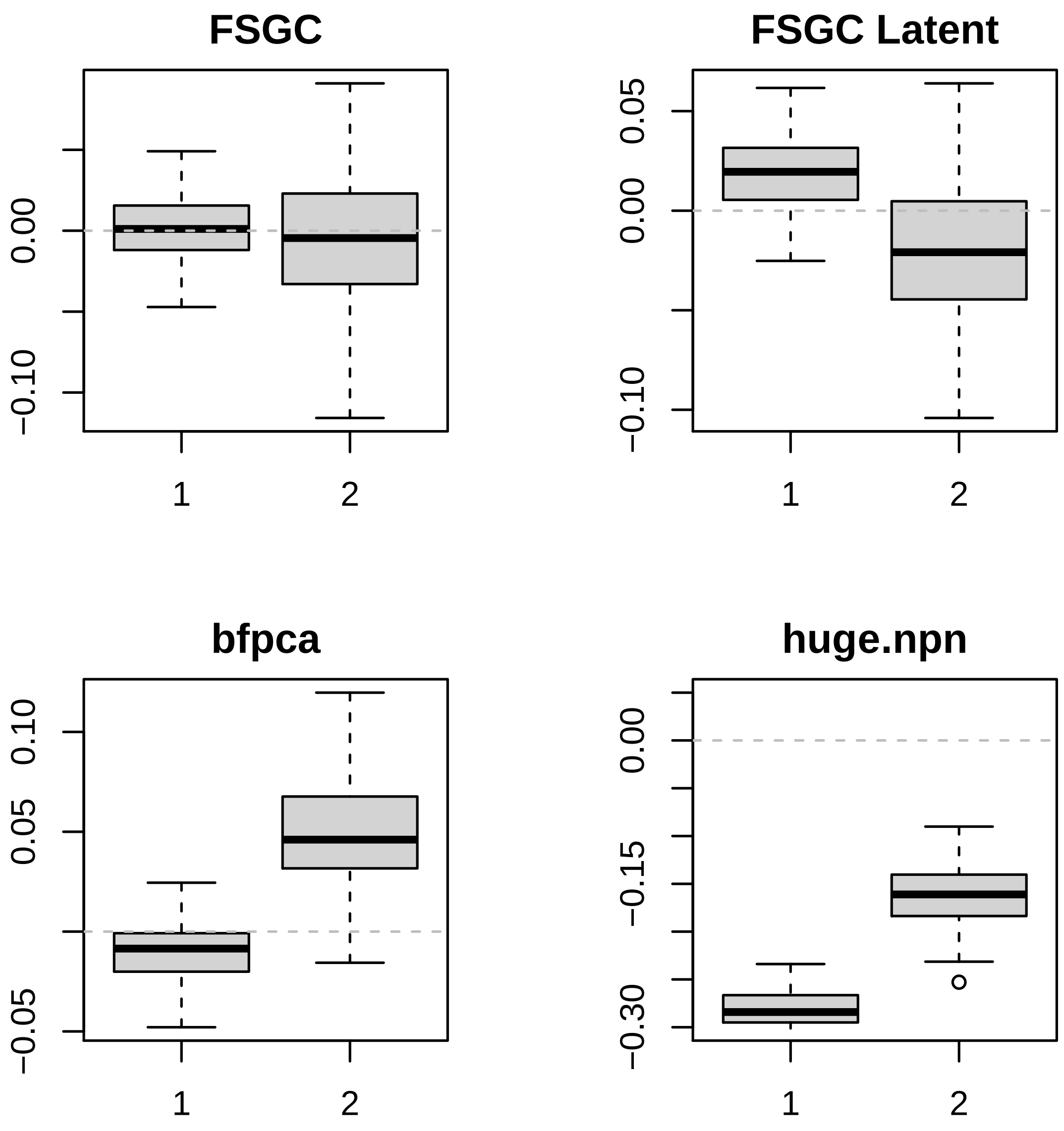

Figure S12: Displayed are the distribution of estimated eigenvalues divided by true eigenvalue -1 ($\frac{\hat{\lambda}_k}{\lambda_k} - 1$), for scenario A, n=500 and non-stationary covariance function.

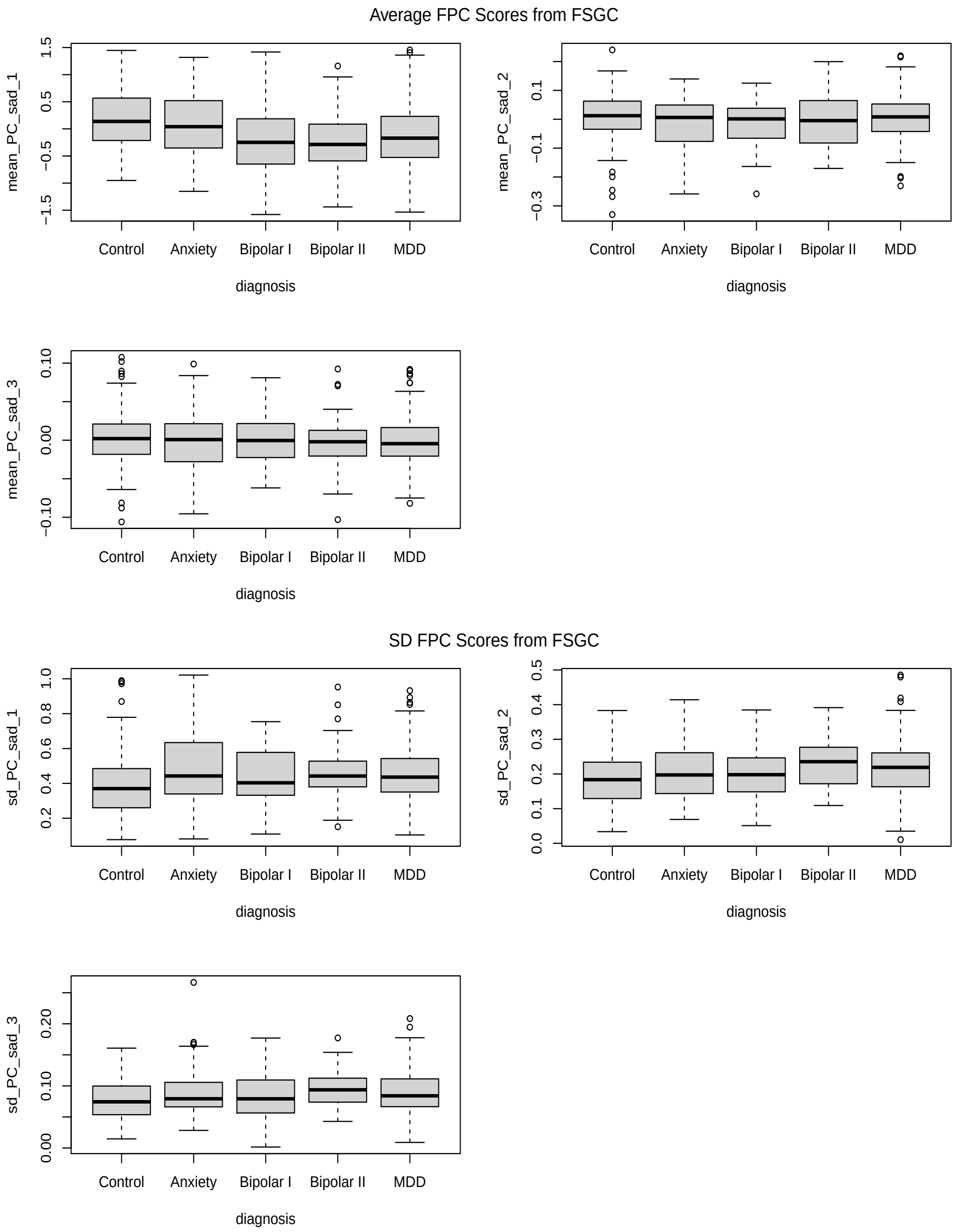

Figure S13: Distribution of mean (top panel) and SD (bottom panel) latent principal component scores of emotional states by the mood disorder groups using the proposed FSGC approach.

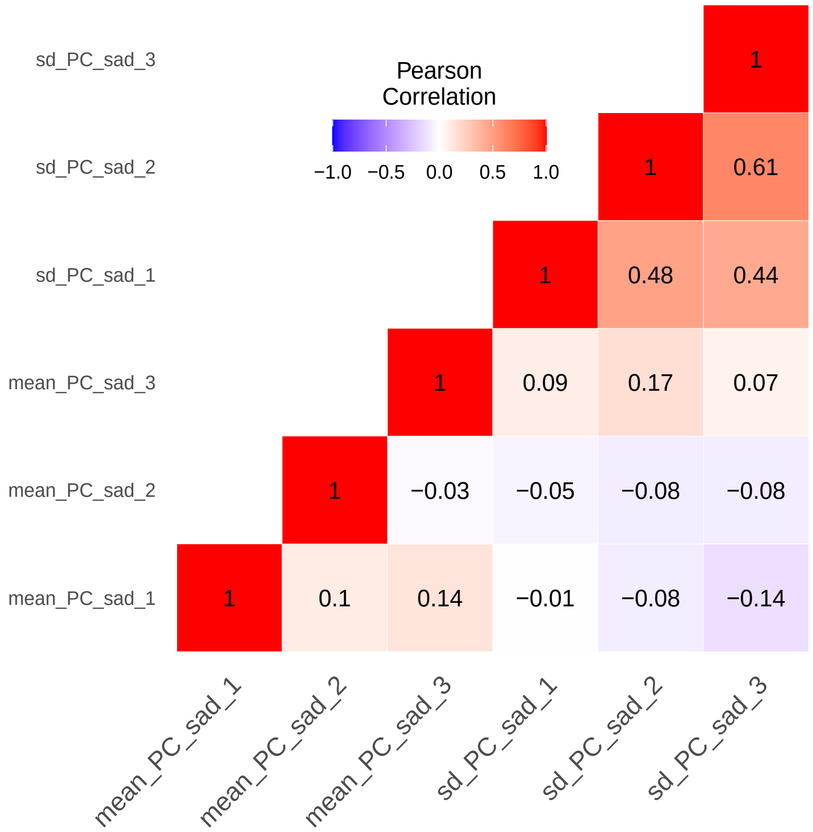

Figure S14: Pearson correlation between mean and SD FPC scores of emotional states.

4 Supplementary Tables

Table S1: Average ISE $\times 10^3$ (and corresponding sd) between true and estimated covariance matrices for different methods and scenarios ($n = 100$). The minimum average ISE is presented in bold-face.

Scenario	Stationary				Non-stationary			
	FSGC	FSGC Latent	Bfpca	Huge.npn	FSGC	FSGC Latent	Bfpca	Huge.npn
A (Binary)	**10 (6)**	12 (7)	32 (18)	29 (13)	8 (6)	**7 (6)**	9 (4)	56 (10)
B (Ordinal)	**5 (4)**	5 (4)	NA	7 (5)	4 (3)	**3 (3)**	NA	6 (3)
C (Truncated)	**9 (9)**	10 (7)	NA	16 (1)	10 (28)	**6 (5)**	NA	50 (9)
D (Continuous)	**1 (1)**	**1 (1)**	NA	**1(1)**	**3 (3)**	**3 (3)**	NA	**3 (3)**

Table S2: Average ISE $\times 10^3$ (and corresponding sd) between true and estimated covariance matrices for different methods and scenarios ($n = 1000$). The minimum average ISE is presented in bold-face.

Scenario	Stationary				Non-stationary			
	FSGC	FSGC Latent	Bfpca	Huge.npn	FSGC	FSGC Latent	Bfpca	Huge.npn
A (Binary)	**1 (1)**	2 (1)	25 (7)	23 (4)	**1 (1)**	**1 (1)**	5 (1)	50 (3)
B (Ordinal)	**1 (0.5)**	1 (1)	NA	3 (1)	0.5 (0.5)	**0.4 (0.4)**	NA	3 (1)
C (Truncated)	**1 (2)**	2 (1)	NA	11 (3)	1 (1)	**1 (0.5)**	NA	43 (3)
D (Continuous)	0.5 (0.4)	**0.4 (0.4)**	NA	0.5(0.4)	0.5 (0.4)	**0.4 (0.3)**	NA	0.4 (0.4)

Table S3: Average ISE $\times 10^3$ (and corresponding sd) between true and estimated covariance matrices for different methods and scenarios, sparse design ($n = 1000$). The minimum average ISE is presented in bold-face.

Scenario	Stationary		Non-stationary	
	FSGC	FSGC Latent	FSGC	FSGC Latent
A (Binary)	**0.5**(2)	1(1)	**1.6**(1)	2(1)
B (Ordinal)	**0.1**(0.1)	0.3(0.1)	**0.7**(0.6)	0.8(0.5)
C (Truncated)	**0.2**(0.1)	0.6(0.3)	**1.6**(0.8)	1.8(0.9)
D (Continuous)	**0.1**(0.04)	**0.1**(0.1)	**0.6** (0.5)	0.8 (0.5)

Table S4: Scenario A, correlation (standard error) between estimated scores from the proposed FSGC (subscript f) and FSGC Latent (subscript l) and estimated scores from true latent curves.

Covariance Type	Sample size	Score1_f	Score1_l	Score2_f	Score2_l
Stationary	n=100	0.870 (0.023)	0.872 (0.022)	0.800 (0.032)	0.801 (0.032)
	n=500	0.875 (0.009)	0.875 (0.009)	0.806 (0.014)	0.806 (0.014)
	n=1000	0.875 (0.007)	0.875 (0.007)	0.807 (0.012)	0.807 (0.012)
Non Stationary	n=100	0.929 (0.021)	0.929 (0.022)	0.896 (0.028)	0.893 (0.029)
	n=500	0.937 (0.006)	0.937 (0.006)	0.905 (0.009)	0.905 (0.009)
	n=1000	0.938 (0.004)	0.938 (0.004)	0.906 (0.006)	0.906 (0.007)

Table S5: $10^3\times$ Average ISE between true and estimated eigenfunctions for different methods in scenario A ($n = 500$). The minimum average ISE is presented in bold-face. For stationary (non stationary) covariance case the first 3 (2) eigenfunctions were used, which explained more than 95% of the variance.

Eigenfunction	Stationary				Non-stationary			
	FSGC	FSGC Latent	Bfpca	Huge.npn	FSGC	FSGC Latent	Bfpca	Huge.npn
1	**14.4**	18.4	55.1	23.2	**0.9**	2.5	3.2	0.9
2	7.3	**3.3**	41.6	55.9	25.6	**0.1**	5.9	77.0
3	**0.3**	3.5	4.5	2.0				

Table S6: Average ISE $\times 10^3$ (and corresponding sd) between true and estimated covariance matrices from the FSGC method for different number of basis functions, dense design ($n = 500$), Scenario A. The result presented in paper (basis=7) is highlighted in bold-face. The performance for an estimator where number of basis was chosen using BIC is given at the bottom.

Number of Basis	Stationary	Non-stationary
	FSGC	FSGC
4	3(1.4)	2.8(1.4)
5	2.5(1.5)	2.5(1.4)
6	2.4(1.5)	2.0(1.4)
7	**2.4(1.5)**	**2.0(1.4)**
8	2.5(1.5)	1.9 (1.4)
9	2.5(1.5)	1.9 (1.4)
BIC chosen	2.6(1.5)	2.0 (1.4)

Table S7: Average (sd) time taken (in seconds) by different estimation methods in dense design ($n = 500$), Scenario A.

Method	Stationary	Non-stationary
Bfpca	135.38 (13.25)	84.61 (5.83)
FSGC (number of basis = 4)	34.6 (3.29)	41.33 (4.22)
FSGC (number of basis = 5)	56.47 (11.25)	53.22 (8.6)
FSGC (number of basis = 7)	76.73 (17.4)	144.78 (36.23)

Table S8: Descriptive statistics for the complete, male and female samples in the real data analysis. For continuous variable the mean and standard deviation is reported, for categorical variable the frequency in each group is mentioned. The P-values are from two-sample t-test and Chi-Square test of association with gender.

Characteristic	Complete (n=497)	Male (n=195)	Female (n=302)	P value
	Mean(sd)	Mean(sd)	Mean(sd)	
Age	41.8 (19.5)	41.2 (21.7)	42.2(17.9)	0.56
Diagnosis: control (N)	134	74	60	0.0001
Diagnosis: Anxiety (N)	97	35	62	
Diagnosis: bipolar I (N)	56	20	36	
Diagnosis: bipolar II (N)	54	22	32	
Diagnosis: MDD (N)	156	44	112	

References

Dey, D. and Zipunnikov, V. (2022), "Semiparametric Gaussian Copula Regression modeling for Mixed Data Types (SGCRM)," *arXiv preprint arXiv:2205.06868*.

Liu, H., Han, F., Yuan, M., Lafferty, J., and Wasserman, L. (2012), "High-dimensional semiparametric Gaussian copula graphical models," *The Annals of Statistics*, 40, 2293–2326.

Yoon, G., Carroll, R. J., and Gaynanova, I. (2018), "Sparse semiparametric canonical correlation analysis for data of mixed types," *arXiv preprint arXiv:1807.05274*.
